# Supplementary figures and images for: Real-world performance of SARS-Cov-2 serology tests in the United States, 2020
Source: PLoS One. 2023 Feb 3;18(2):e0279956. doi: 10.1371/journal.pone.0279956 (PMC9897562; doi:10.1371/journal.pone.0279956)

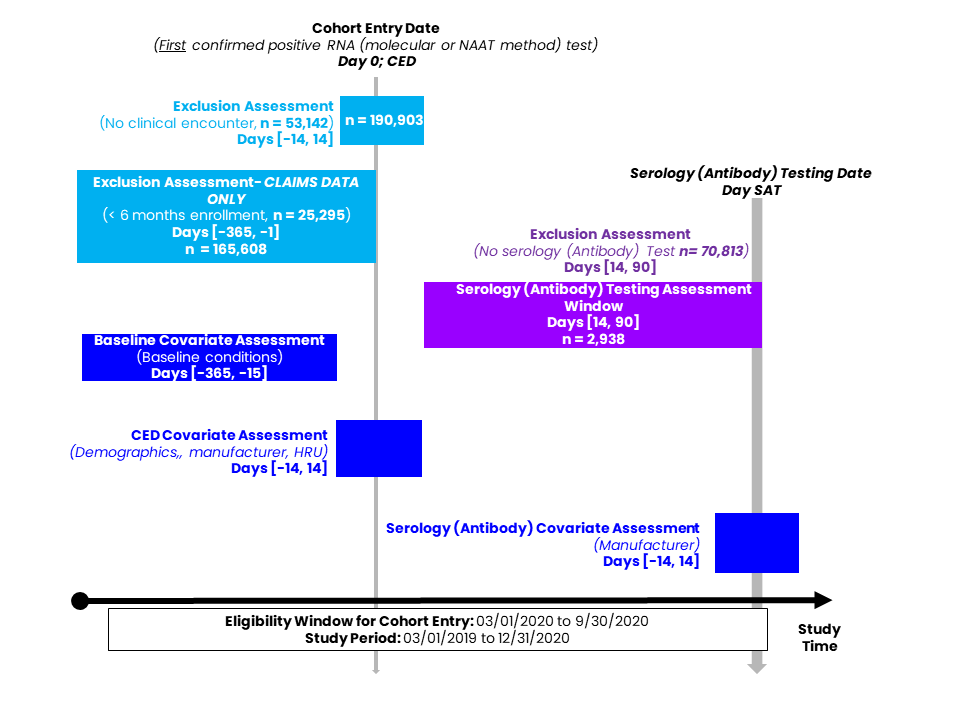

Supplement: S1 Fig — (TIF) [file pone.0279956.s001.tif]

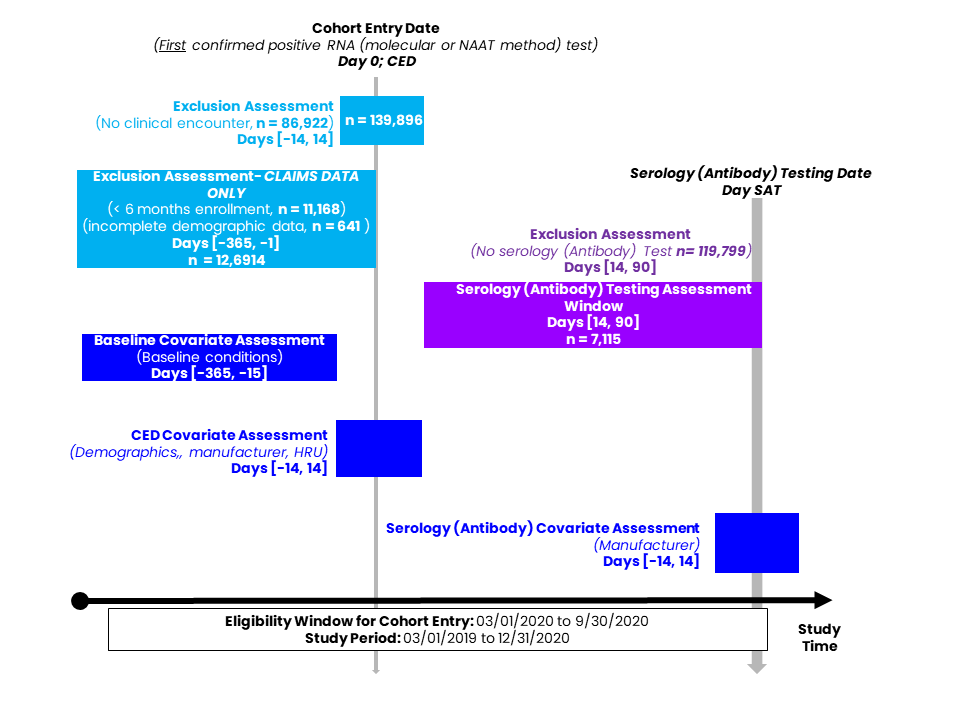

Supplement: S2 Fig — (TIF) [file pone.0279956.s002.tif]

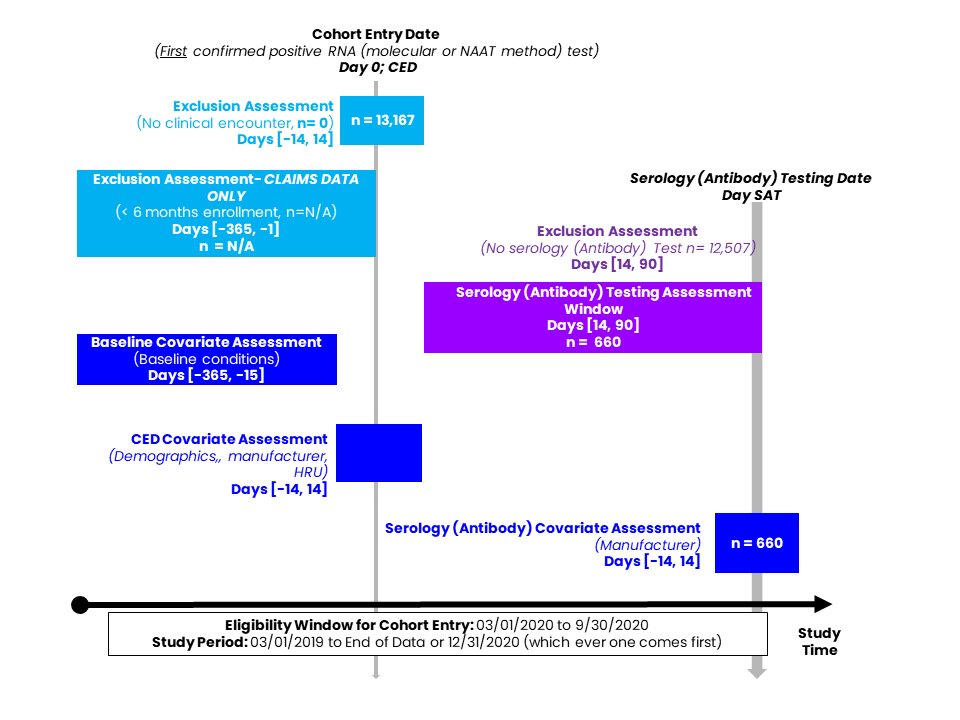

Supplement: S3 Fig — (TIF) [file pone.0279956.s003.tif]

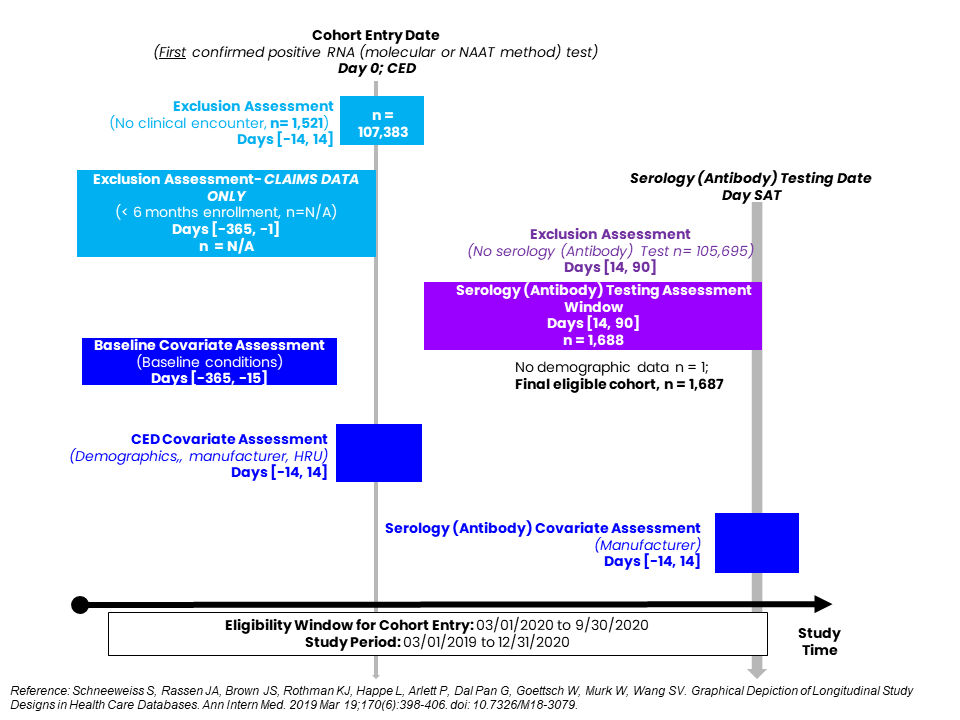

Supplement: S4 Fig — (TIF) [file pone.0279956.s004.tif]

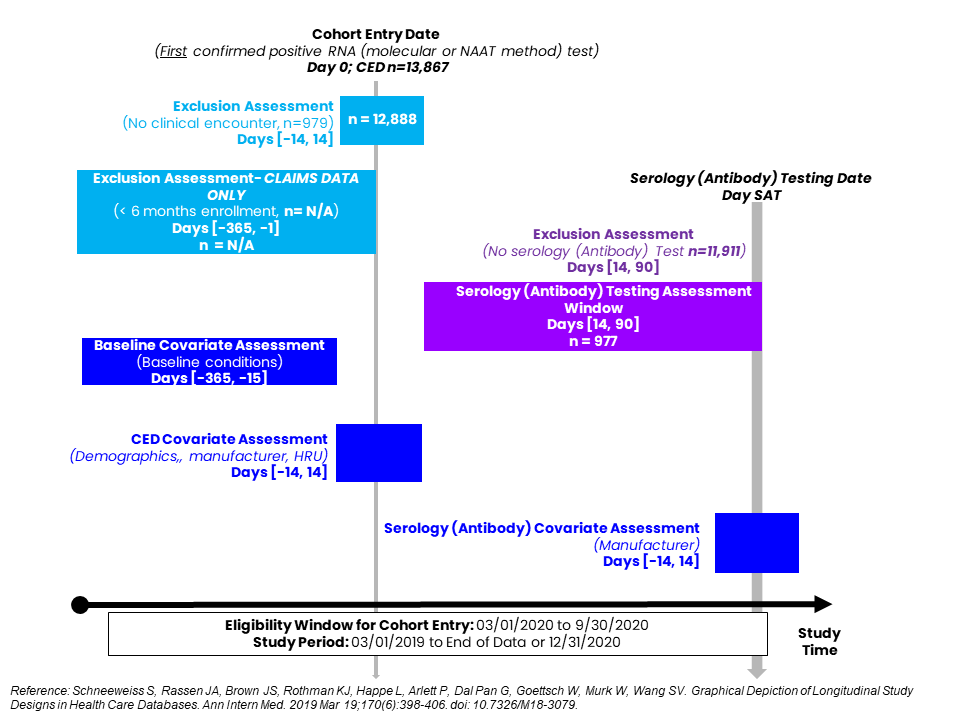

Supplement: S5 Fig — (TIF) [file pone.0279956.s005.tif]

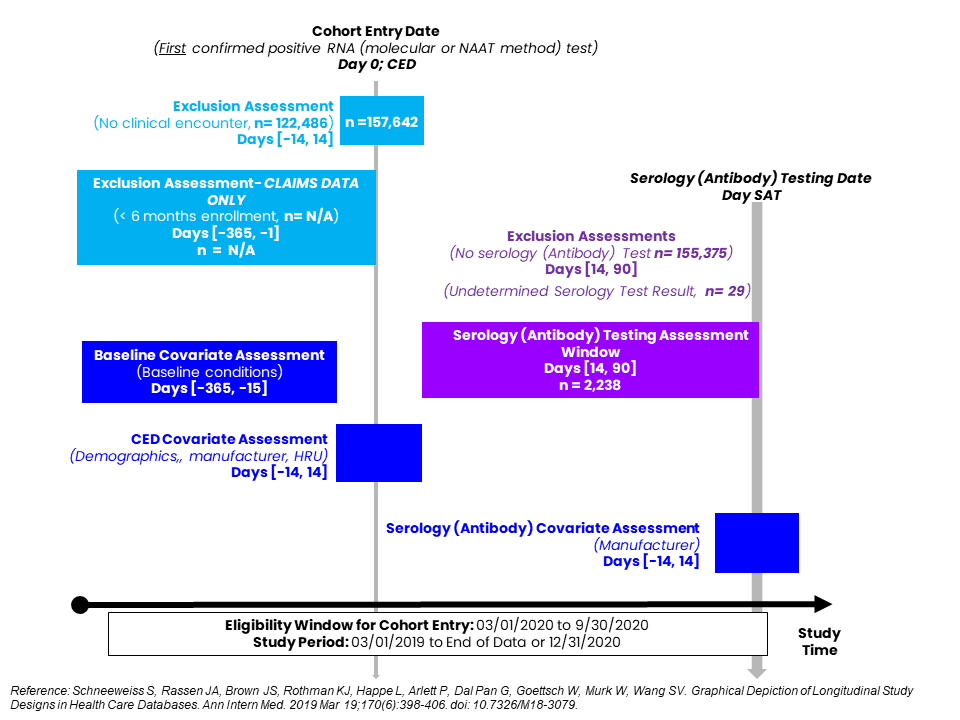

Supplement: S6 Fig — (TIF) [file pone.0279956.s006.tif]
