# Supplementary material for: Real-world performance of SARS-Cov-2 serology tests in the United States, 2020
Source: PLoS One. 2023 Feb 3;18(2):e0279956. doi: 10.1371/journal.pone.0279956 (PMC9897562; doi:10.1371/journal.pone.0279956)
Supplement: S1 Table — (DOCX) [file pone.0279956.s007.docx]

S1 Table: Characteristics of participating data sources and representative populations

|  | **A** | **B** | **C** | **D** | **F** | **E** |
| --- | --- | --- | --- | --- | --- | --- |
| **Data source** | (HealthVerity) 01/01/2018 – 10/05/2020 (Random Data Sample comprised of administrative medical and pharmacy claims data, EMR, lab and hospital chargemaster data) | Administrative claims data from a single, large, US insurer | Electronic Health Records | Health Catalyst EHR-based Electronic Data Warehouse | EHR (EPIC) and Lab system (SOFT) | CoRDaCo (COVID-19 Research Data Commons) |
| **Period data collected** | 03/01/2020- 12/31/2020 | 03/09/2020- 12/16/2020 | 03/01/2020 - 1/29/2021 | 01/01/ 2020 -11/20/2020 | 03/01/2020 - 1/29/2021 | 03/01/2019 -02/11/2021 |
| **States represented in the study population** | 50 States | 50 States | California | Arizona, California, Colorado,  Alaska,  Delaware,  Hawaii,  Iowa,  Illinois,  Indiana,  Kansas,  Kentucky, Louisiana, Michigan, Montana,  N Carolina, Nebraska,  Nevada,  Ohio,  Oklahoma, Wisconsin, Wyoming | Alabama, Alaska, Arizona, Arkansas, California, Colorado, Delaware, Florida, Georgia, Hawaii, Idaho, Illinois, Indiana,  Iowa,  Kansas, Kentucky, Louisiana, Maryland, Massachusett, Michigan, Minnesota, Mississippi, Missouri, Montana,  Nebraska, Nevada,  New Jersey,  New Mexico,  New York,  N Carolina,  N Dakota,  Ohio, Oklahoma, Oregon, Pennsylvania, S Carolina,  S Dakota, Tennessee, Texas,  Utah, Virginia, Washington, Wisconsin, Wyoming | Alabama,  Arizona,  Arkansas,  California,  Colorado,  District of Columbia,  Florida,  Georgia,  Illinois,  Indiana^2^,  Iowa,  Kansas,  Kentucky,  Louisiana,  Maryland,  Michigan,  Minnesota, Mississippi,  Missouri,  N. Carolina, Nebraska,  Nevada,  New York,  Ohio,  Oklahoma,  Oregon, Pennsylvania,  S. Carolina, Tennessee,  Texas,  Virginia,  W. Virginia, Washington, Wisconsin,  Wyoming |
| **Data processing & Data Integrity:** | The contributing vendor's data aggregated by HealthVerity in their HealthVerity Marketplace is monitored throughout processing to ensure all rows sent by the vendor are received. This is done by total record count and key fields. Data is also trended on those same key metrics to ensure the vendor sent full transmissions. The HealthVerity data used for the analysis was uploaded into the Aetion Evidence Platform, which was used to perform all the analyses. The platform is a data-handling technology, which allows for the analysis of large patient datasets by indexing patient data into a form that can be queried by an internal patient variable language. Data is minimally transformed at the point of connection to the Aetion Evidence Platform, thus the original format of the raw HealthVerity data is preserved. At the point of data connection to the platform, some discard rules are applied. Patients are excluded if the patient ID is missing. Patient events are excluded if there are no dates associated with them, or if the start date of the event is preceded by the end date of the event (e.g. discharge date precedes admission date for an inpatient event). Aetion IDs are assigned to HealthVerity patient IDs (HVIDs) and a crosswalk file is kept as a protected file. | **Standardization of Data Entry and Data Structure:** Medical and pharmacy claims data are captured, predominantly electronically, from sites of care-seeking third-party reimbursement for both Medicare and commercial plans using the industry-standard data collection forms HCFA/CMS-1500 for facility claims, UB04/CMS-1450 for professional services and outpatient claims, and NCPDP for pharmacy claims or their electronic equivalents. Structured data from these standardized forms are coded using the International Classification of Diseases, Tenth Revision, Clinical Modification (ICD-10-CM), National Drug Codes (NDC), Current Procedural Terminology (CPT) codes, and Logical Observation Identifiers Names and Codes (LOINC) codes, and Diagnosis Related Groups (DRG). This nomenclature ensures consistency of data collection across geographic regions, health systems, and payers throughout the United States. **Methods to Control for Errors in Sampling and Data Collection:** Claims that do not adhere to the form or coding standards described above are rejected from reimbursement, minimizing the risk that inappropriately structured data are included in the database. | Electronic health records (EHRs) of over 300,000 individuals from 20 professional schools, 6 academic health centers, and 12 hospitals across the University of California Health (UC Health) receiving COVID related care were de-identified and processed using OMOP common data model into UC COVID Research Database (UC CORDS). The standard OMOP guidelines were followed to assess the quality of the data and maintain integrity. | Data from 19 health systems are ingested into Health Catalyst's Data Operating System, aggregated, normalized across data sources, and stored in Health Catalyst's internal common data model format in a centralized data repository. With each data refresh, standard internal quality assurance steps are performed including assessment of primary keys, date ranges, and volumetric assessments (row counts, patient counts, etc.) to ensure data integrity, characterize potential data loss, and perform remediation steps when appropriate and feasible. | Lab results are compiled from the EHR (EPIC) by our Department of Data and Analytics and populate distinctly, curated COVID-19 PCR and Serology tables in a DB2 environment. To link with lab manufacturer data in our SOFT LIS environment, Mayo has set up an MS SQL database that mirrors all available COVID testing data from the LIS. We have successfully queried the database to acquire manufacturer data on all COVID patients from our EHR, but ongoing validation is being conducted to confirm and improve linkage between these previously disparate data environments. | The original data comes from the Indiana Network of Patient Care (INPC) - one of the largest health information exchanges in the nation and contains records from health systems across the state. CoRDaCo uses MDClone software to scrub sensitive patient information and supplemented it with info such as age, mobility, and other social determinants of health. RI and IU’s University Information Technology Services (UITS) Scalable Compute Archive (RT-SCA) uses curated datasets of COVID-19 patient data to generate synthetic medical data. Synthetic data reflects the characteristics of real patient data but does not include real patient information. Because it is statistically similar, it can be used in the same way as real data, but without compromising privacy. |

1. University of California Health.
2. Majority (99.14%) of the patients in the study population were Indiana Residents.
