# Supplementary material for: Real-world performance of SARS-Cov-2 serology tests in the United States, 2020
Source: PLoS One. 2023 Feb 3;18(2):e0279956. doi: 10.1371/journal.pone.0279956 (PMC9897562; doi:10.1371/journal.pone.0279956)
Supplement: S2 Table — (DOCX) [file pone.0279956.s008.docx]

# S2 Table. Phenotype (code-lists) for specified presenting symptoms & pre-existing conditions.

**Table 1: +RNA Identification**

| **Variable** | **Algorithm / Definitions** | **Setting^1^** | **Source** | **Reference** |
| --- | --- | --- | --- | --- |
| Tested | LOINC: 94500-6, 94309-2  CPT/HCPCS: 87635, U0001, U0002  Note: additional CPT/LOINC codes will be added to variable definition as new codes are released. | IP, OP, ED, or GL | Claims or EHR |  |
| Untested | No occurrence of LOINCs 94500-6, 94309-2 AND  No occurrence of CPT/HCPCS 87635, U0001, U0002  Note: additional CPT/LOINC codes will be added to variable definition as new codes are released. | IP, OP, ED, or GL | Claims or EHR |  |
| COVID-19 positive | LOINC: 94500-6, 94309-2 AND  Result: Positive for 2019-nCoV, Presumptive Positive for 2019-nCoV | IP, OP, ED, or GL | Claims or EHR |  |
| COVID-19 negative/not detected | LOINC: 94500-6, 94309-2 AND  Result: Not detected | IP, OP, ED, or GL | Claims or EHR |  |
| Unknown/results pending | LOINC: 94500-6, 94309-2 AND  No result recorded other result recorded | IP, OP, ED, or GL | Claims or EHR |  |

1. IP=Inpatient; OP=Outpatient; ED=Emergency Department; GL=Group Living Facility

**Table 2: Pre-Existing Conditions Definitions^1^**

| **Variable** | **Algorithm / Definitions** | **Setting^2^** | **Source** | **Reference** |
| --- | --- | --- | --- | --- |
| **Significant comorbidities** |  |  |  |  |
| Asthma | ICD-10-CM: J45.90, J45.21, J45.22, J45.31, J45.4, J45.41, J45.42, J45.51, J45.902, J45.909, J45.3, J45.30, J45.32, J45.5, J45.991, J45, J45.20, J45.50, J45.99, J45.2, J45.40, J45.52, J45.9, J45.901, J45.990, J45.998 | IP, OP, ED, or GL | Claims or EHR |  |
| Cancer | ICD-10-CM: C00, C00.0, C00.1, C00.2, C00.3, C00.4, C00.5, C00.6, C00.8, C00.9, C01, C02, C02.0, C02.1, C02.2, C02.3, C02.4, C02.8, C02.9, C03, C03.0, C03.1, C03.9, C04, C04.0, C04.1, C04.8, C04.9, C05, C05.0, C05.1, C05.2, C05.8, C05.9, C06, C06.0, C06.1, C06.2, C06.8, C06.80, C06.89, C06.9, C07, C08, C08.0, C08.1, C08.9, C09, C09.0, C09.1, C09.8, C09.9, C10, C10.0, C10.1, C10.2, C10.3, C10.4, C10.8, C10.9, C11, C11.0, C11.1, C11.2, C11.3, C11.8, C11.9, C12, C13, C13.0, C13.1, C13.2, C13.8, C13.9, C14, C14.0, C14.2, C14.8, C15, C15.3, C15.4, C15.5, C15.8, C15.9, C16, C16.0, C16.1, C16.2, C16.3, C16.4, C16.5, C16.6, C16.8, C16.9, C17, C17.0, C17.1, C17.2, C17.3, C17.8, C17.9, C18, C18.0, C18.1, C18.2, C18.3, C18.4, C18.5, C18.6, C18.7, C18.8, C18.9, C19, C20, C21, C21.0, C21.1, C21.2, C21.8, C22, C22.0, C22.1, C22.2, C22.3, C22.4, C22.7, C22.8, C22.9, C23, C24, C24.0, C24.1, C24.8, C24.9, C25, C25.0, C25.1, C25.2, C25.3, C25.4, C25.7, C25.8, C25.9, C26, C26.0, C26.1, C26.9, C30, C30.0, C30.1, C31, C31.0, C31.1, C31.2, C31.3, C31.8, C31.9, C32, C32.0, C32.1, C32.2, C32.3, C32.8, C32.9, C33, C34, C34.0, C34.00, C34.01, C34.02, C34.1, C34.10, C34.11, C34.12, C34.2, C34.3, C34.30, C34.31, C34.32, C34.8, C34.80, C34.81, C34.82, C34.9, C34.90, C34.91, C34.92, C37, C38, C38.0, C38.1, C38.2, C38.3, C38.4, C38.8, C39, C39.0, C39.9, C40, C40.0, C40.00, C40.01, C40.02, C40.1, C40.10, C40.11, C40.12, C40.2, C40.20, C40.21, C40.22, C40.3, C40.30, C40.31, C40.32, C40.8, C40.80, C40.81, C40.82, C40.9, C40.90, C40.91, C40.92, C41, C41.0, C41.1, C41.2, C41.3, C41.4, C41.9, C43, C43.0, C43.1, C43.10, C43.11, C43.111, C43.112, C43.12, C43.121, C43.122, C43.2, C43.20, C43.21, C43.22, C43.3, C43.30, C43.31, C43.39, C43.4, C43.5, C43.51, C43.52, C43.59, C43.6, C43.60, C43.61, C43.62, C43.7, C43.70, C43.71, C43.72, C43.8, C43.9, C45, C45.0, C45.1, C45.2, C45.7, C45.9, C46, C46.0, C46.1, C46.2, C46.3, C46.4, C46.5, C46.50, C46.51, C46.52, C46.7, C46.9, C47, C47.0, C47.1, C47.10, C47.11, C47.12, C47.2, C47.20, C47.21, C47.22, C47.3, C47.4, C47.5, C47.6, C47.8, C47.9, C48, C48.0, C48.1, C48.2, C48.8, C49, C49.0, C49.1, C49.10, C49.11, C49.12, C49.2, C49.20, C49.21, C49.22, C49.3, C49.4, C49.5, C49.6, C49.8, C49.9, C49.A, C49.A0, C49.A1, C49.A2, C49.A3, C49.A4, C49.A5, C49.A9, C50, C50.0, C50.01, C50.011, C50.012, C50.019, C50.02, C50.021, C50.022, C50.029, C50.1, C50.11, C50.111, C50.112, C50.119, C50.12, C50.121, C50.122, C50.129, C50.2, C50.21, C50.211, C50.212, C50.219, C50.22, C50.221, C50.222, C50.229, C50.3, C50.31, C50.311, C50.312, C50.319, C50.32, C50.321, C50.322, C50.329, C50.4, C50.41, C50.411, C50.412, C50.419, C50.42, C50.421, C50.422, C50.429, C50.5, C50.51, C50.511, C50.512, C50.519, C50.52, C50.521, C50.522, C50.529, C50.6, C50.61, C50.611, C50.612, C50.619, C50.62, C50.621, C50.622, C50.629, C50.8, C50.81, C50.811, C50.812, C50.819, C50.82, C50.821, C50.822, C50.829, C50.9, C50.91, C50.911, C50.912, C50.919, C50.92, C50.921, C50.922, C50.929, C51, C51.0, C51.1, C51.2, C51.8, C51.9, C52, C53, C53.0, C53.1, C53.8, C53.9, C54, C54.0, C54.1, C54.2, C54.3, C54.8, C54.9, C55, C56, C56.1, C56.2, C56.9, C57, C57.0, C57.00, C57.01, C57.02, C57.1, C57.10, C57.11, C57.12, C57.2, C57.20, C57.21, C57.22, C57.3, C57.4, C57.7, C57.8, C57.9, C58, C60, C60.0, C60.1, C60.2, C60.8, C60.9, C61, C62, C62.0, C62.00, C62.01, C62.02, C62.1, C62.10, C62.11, C62.12, C62.9, C62.90, C62.91, C62.92, C63, C63.0, C63.00, C63.01, C63.02, C63.1, C63.10, C63.11, C63.12, C63.2, C63.7, C63.8, C63.9, C64, C64.1, C64.2, C64.9, C65, C65.1, C65.2, C65.9, C66, C66.1, C66.2, C66.9, C67, C67.0, C67.1, C67.2, C67.3, C67.4, C67.5, C67.6, C67.7, C67.8, C67.9, C68, C68.0, C68.1, C68.8, C68.9, C69, C69.0, C69.00, C69.01, C69.02, C69.1, C69.10, C69.11, C69.12, C69.2, C69.20, C69.21, C69.22, C69.3, C69.30, C69.31, C69.32, C69.4, C69.40, C69.41, C69.42, C69.5, C69.50, C69.51, C69.52, C69.6, C69.60, C69.61, C69.62, C69.8, C69.80, C69.81, C69.82, C69.9, C69.90, C69.91, C69.92, C70, C70.0, C70.1, C70.9, C71, C71.0, C71.1, C71.2, C71.3, C71.4, C71.5, C71.6, C71.7, C71.8, C71.9, C72, C72.0, C72.1, C72.2, C72.20, C72.21, C72.22, C72.3, C72.30, C72.31, C72.32, C72.4, C72.40, C72.41, C72.42, C72.5, C72.50, C72.59, C72.9, C73, C74, C74.0, C74.00, C74.01, C74.02, C74.1, C74.10, C74.11, C74.12, C74.9, C74.90, C74.91, C74.92, C75, C75.0, C75.1, C75.2, C75.3, C75.4, C75.5, C75.8, C75.9, C76, C76.0, C76.1, C76.2, C76.3, C76.4, C76.40, C76.41, C76.42, C76.5, C76.50, C76.51, C76.52, C76.8, C7A, C7A.0, C7A.00, C7A.01, C7A.010, C7A.011, C7A.012, C7A.019, C7A.02, C7A.020, C7A.021, C7A.022, C7A.023, C7A.024, C7A.025, C7A.026, C7A.029, C7A.09, C7A.090, C7A.091, C7A.092, C7A.093, C7A.094, C7A.095, C7A.096, C7A.098, C7A.1, C7A.8, C7B, C7B.0, C7B.00, C7B.01, C7B.02, C7B.03, C7B.04, C7B.09, C7B.1, C7B.8, C81, C81.0, C81.00, C81.01, C81.02, C81.03, C81.04, C81.05, C81.06, C81.07, C81.08, C81.09, C81.1, C81.10, C81.11, C81.12, C81.13, C81.14, C81.15, C81.16, C81.17, C81.18, C81.19, C81.2, C81.20, C81.21, C81.22, C81.23, C81.24, C81.25, C81.26, C81.27, C81.28, C81.29, C81.3, C81.30, C81.31, C81.32, C81.33, C81.34, C81.35, C81.36, C81.37, C81.38, C81.39, C81.4, C81.40, C81.41, C81.42, C81.43, C81.44, C81.45, C81.46, C81.47, C81.48, C81.49, C81.7, C81.70, C81.71, C81.72, C81.73, C81.74, C81.75, C81.76, C81.77, C81.78, C81.79, C81.9, C81.90, C81.91, C81.92, C81.93, C81.94, C81.95, C81.96, C81.97, C81.98, C81.99, C82, C82.0, C82.00, C82.01, C82.02, C82.03, C82.04, C82.05, C82.06, C82.07, C82.08, C82.09, C82.1, C82.10, C82.11, C82.12, C82.13, C82.14, C82.15, C82.16, C82.17, C82.18, C82.19, C82.2, C82.20, C82.21, C82.22, C82.23, C82.24, C82.25, C82.26, C82.27, C82.28, C82.29, C82.3, C82.30, C82.31, C82.32, C82.33, C82.34, C82.35, C82.36, C82.37, C82.38, C82.39, C82.4, C82.40, C82.41, C82.42, C82.43, C82.44, C82.45, C82.46, C82.47, C82.48, C82.49, C82.5, C82.50, C82.51, C82.52, C82.53, C82.54, C82.55, C82.56, C82.57, C82.58, C82.59, C82.6, C82.60, C82.61, C82.62, C82.63, C82.64, C82.65, C82.66, C82.67, C82.68, C82.69, C82.8, C82.80, C82.81, C82.82, C82.83, C82.84, C82.85, C82.86, C82.87, C82.88, C82.89, C82.9, C82.90, C82.91, C82.92, C82.93, C82.94, C82.95, C82.96, C82.97, C82.98, C82.99, C83, C83.0, C83.00, C83.01, C83.02, C83.03, C83.04, C83.05, C83.06, C83.07, C83.08, C83.09, C83.1, C83.10, C83.11, C83.12, C83.13, C83.14, C83.15, C83.16, C83.17, C83.18, C83.19, C83.3, C83.30, C83.31, C83.32, C83.33, C83.34, C83.35, C83.36, C83.37, C83.38, C83.39, C83.5, C83.50, C83.51, C83.52, C83.53, C83.54, C83.55, C83.56, C83.57, C83.58, C83.59, C83.7, C83.70, C83.71, C83.72, C83.73, C83.74, C83.75, C83.76, C83.77, C83.78, C83.79, C83.8, C83.80, C83.81, C83.82, C83.83, C83.84, C83.85, C83.86, C83.87, C83.88, C83.89, C83.9, C83.90, C83.91, C83.92, C83.93, C83.94, C83.95, C83.96, C83.97, C83.98, C83.99, C84, C84.0, C84.00, C84.01, C84.02, C84.03, C84.04, C84.05, C84.06, C84.07, C84.08, C84.09, C84.1, C84.10, C84.11, C84.12, C84.13, C84.14, C84.15, C84.16, C84.17, C84.18, C84.19, C84.4, C84.40, C84.41, C84.42, C84.43, C84.44, C84.45, C84.46, C84.47, C84.48, C84.49, C84.6, C84.60, C84.61, C84.62, C84.63, C84.64, C84.65, C84.66, C84.67, C84.68, C84.69, C84.7, C84.70, C84.71, C84.72, C84.73, C84.74, C84.75, C84.76, C84.77, C84.78, C84.79, C84.9, C84.90, C84.91, C84.92, C84.93, C84.94, C84.95, C84.96, C84.97, C84.98, C84.99, C84.A, C84.A0, C84.A1, C84.A2, C84.A3, C84.A4, C84.A5, C84.A6, C84.A7, C84.A8, C84.A9, C84.Z, C84.Z0, C84.Z1, C84.Z2, C84.Z3, C84.Z4, C84.Z5, C84.Z6, C84.Z7, C84.Z8, C84.Z9, C85, C85.1, C85.10, C85.11, C85.12, C85.13, C85.14, C85.15, C85.16, C85.17, C85.18, C85.19, C85.2, C85.20, C85.21, C85.22, C85.23, C85.24, C85.25, C85.26, C85.27, C85.28, C85.29, C85.8, C85.80, C85.81, C85.82, C85.83, C85.84, C85.85, C85.86, C85.87, C85.88, C85.89, C85.9, C85.90, C85.91, C85.92, C85.93, C85.94, C85.95, C85.96, C85.97, C85.98, C85.99, C88, C88.0, C88.2, C88.3, C88.4, C88.8, C88.9, C90, C90.0, C90.00, C90.01, C90.02, C90.1, C90.10, C90.11, C90.12, C90.2, C90.20, C90.21, C90.22, C90.3, C90.30, C90.31, C90.32, C91, C91.0, C91.00, C91.01, C91.02, C91.1, C91.10, C91.11, C91.12, C91.3, C91.30, C91.31, C91.32, C91.4, C91.40, C91.41, C91.42, C91.5, C91.50, C91.51, C91.52, C91.6, C91.60, C91.61, C91.62, C91.9, C91.90, C91.91, C91.92, C91.A, C91.A0, C91.A1, C91.A2, C91.Z, C91.Z0, C91.Z1, C91.Z2, C92, C92.0, C92.00, C92.01, C92.02, C92.1, C92.10, C92.11, C92.12, C92.2, C92.20, C92.21, C92.22, C92.3, C92.30, C92.31, C92.32, C92.4, C92.40, C92.41, C92.42, C92.5, C92.50, C92.51, C92.52, C92.6, C92.60, C92.61, C92.62, C92.9, C92.90, C92.91, C92.92, C92.A, C92.A0, C92.A1, C92.A2, C92.Z, C92.Z0, C92.Z1, C92.Z2, C93, C93.0, C93.00, C93.01, C93.02, C93.1, C93.10, C93.11, C93.12, C93.3, C93.30, C93.31, C93.32, C93.9, C93.90, C93.91, C93.92, C93.Z, C93.Z0, C93.Z1, C93.Z2, C94, C94.0, C94.00, C94.01, C94.02, C94.2, C94.20, C94.21, C94.22, C94.3, C94.30, C94.31, C94.32, C94.4, C94.40, C94.41, C94.42, C94.6, C94.8, C94.80, C94.81, C94.82, C95, C95.0, C95.00, C95.01, C95.02, C95.1, C95.10, C95.11, C95.12, C95.9, C95.90, C95.91, C95.92, C96, C96.0, C96.2, C96.20, C96.21, C96.22, C96.29, C96.4, C96.5, C96.6, C96.9, C96.A, C96.Z, C77, C77.0, C77.1, C77.2, C77.3, C77.4, C77.5, C77.8, C77.9, C78, C78.0, C78.00, C78.01, C78.02, C78.1, C78.2, C78.3, C78.30, C78.39, C78.4, C78.5, C78.6, C78.7, C78.8, C78.80, C78.89, C79, C79.0, C79.00, C79.01, C79.02, C79.1, C79.10, C79.11, C79.19, C79.2, C79.3, C79.31, C79.32, C79.4, C79.40, C79.49, C79.5, C79.51, C79.52, C79.6, C79.60, C79.61, C79.62, C79.7, C79.70, C79.71, C79.72, C79.8, C79.81, C79.82, C79.89, C79.9, C80, C80.0, C80.1, C80.2 | IP, OP, ED, or GL | Claims or EHR | [Quan et al, 2005](https://pubmed.ncbi.nlm.nih.gov/16224307/) |
| Chronic lung disease | ICD-10-CM: I27.8*, I27.9*, J40*–J47*, J60*–J67*, J68.4, J70.1, J70.3 | IP, OP, ED, or GL | Claims or EHR | [Quan et al, 2005](https://pubmed.ncbi.nlm.nih.gov/16224307/) |
| Cardiovascular disease (any) | ICD-10-CM: I00*-I99* | IP, OP, ED, or GL | Claims or EHR | [Nyström et al, 2017](https://www.sciencedirect.com/science/article/pii/S0168822716304429#s0095) |
| …Coronary Artery Disease | ICD-10-CM: I20.0, I21.0, I21.01, I21.02, I21.09, I21.1, I21.11, I21.19, I21.2, I21.21, I21.29, I21.3, I21.4, I22.0, I22.1, I22.2, I22.8, I22.9, I24, I24.0, I24.1, I24.8, I24.9, I25.1, I25.2, I25.8, I25.9, I21, I21.9, I21.A1, I21.A9, I22, I24.4, I25.10, I25.11, I25.110, I25.111, I25.118, I25.119, I25.18, I25.81, I25.810, I25.811, I25.812, I25.82, I25.83, I25.84, I25.89, | IP, OP, ED, or GL | Claims or EHR |  |
| …Congestive Heart Failure | ICD-10-CM: I09.9, I11.0, I13.0, I13.2, I25.5, I42.0, I42.5 – I42.9, I43*, I50* | IP, OP, ED, or GL | Claims or EHR | [So et al, 2006](https://www.ncbi.nlm.nih.gov/pmc/articles/PMC1781440/) |
| …AMI | ICD-10-CM: I21.09, I21.19, I21.11, I21.29, I21.4, I21.3 | IP, OP, ED, or GL | IP, OP, ED, or GL | [Sentinel Initiative](https://www.sentinelinitiative.org/about/how-sentinel-gets-its-data),  [Fung, K. W., et al. (2016)](https://www.ncbi.nlm.nih.gov/pmc/articles/PMC4862764/) |
| Stroke | ICD-10-CM: I63.22, I63.139, I63.239, I63.019, I63.119, I63.219, I63.59, I63.20, I63.30, I63.40, I63.50, I67.8, I63.00, I63.011, I63.012, I63.013, I63.0, I63.031, I63.032, I63.033, I63.039, I63.0, I63.10, I63.111, I63.112, I63.113, I63.12, I63.131, I63.132, I63.133, I63.19, I63.211, I63.212, I63.213, I63.231, I63.232, I63.233, I63.29, I63.311, I63.312, I63.313, I63.319, I63.321, I63.322, I63.323, I63.329, I63.331, I63.332, I63.333, I63.339, I63.341, I63.342, I63.343, I63.349, I63.39, I63.411, I63.412, I63.413, I63.419, I63.422, I63.423, I63.429, I63.431, I63.432, I63.433, I63.439, I63.441, I63.442, I63.443, I63.449, I63.49, I63.511, I63.512, I63.513, I63.519, I63.521,I63.522, I63.523, I63.529, I63.531, I63.532, I63.533, I63.539, I63.541, I63.542, I63.543, I63.549, I63.6, I63.8, I63.9 | IP, OP, ED, or GL | IP, OP, ED, or GL | [Sentinel Initiative](https://www.sentinelinitiative.org/about/how-sentinel-gets-its-data),  [Fung, K. W., et al. (2016)](https://www.ncbi.nlm.nih.gov/pmc/articles/PMC4862764/) |
| Prior ACS, Coronary Revascularization | ICD-10-CM: G45*, G46*, H34.0*, I60*– I62*, I64*-I69*, I20.0, I24.9, I22*, I25.2, I25.812, I25.810  ICD-10-PCS: 021008F, 0210098, 021009F, 02100AW, 02100J8, 0210344, 021049W, 02104J3, 02104J8, 0211088, 021109F, 02110A9, 02110JF, 02110K8, 02110K9, 02110Z3, 02113D4, 0211499, 021149W, 02114J9, 02114JC, 02114K3, 02114KF, 02114KW, 02114Z8, 0212088, 021209C, 021209W, 02120JW, 02120K8, 02120KW, 02120Z8, 021248F, 021249C, 021249F, 021249W, 02124JC, 02124JW, 02124K8, 02124KW, 02124Z8, 0213088, 021308C, 021308W, 0213093, 0213098, 02130J3, 02130KF, 0213444, 0213493, 02134JF, 02134KC, 02134KF, 02134KW, 027006Z, 027007Z, 02700E6, 0270346, 0270356, 02703D6, 02703F6, 02703GZ, 02703TZ, 02703Z6, 027045Z, 027047Z, 02704DZ, 02710E6, 02710FZ, 02710G6, 0271346, 02713FZ, 02713Z6, 02713ZZ, 027145Z, 02714DZ, 02714EZ, 02714FZ, 02714TZ, 0272056, 0272066, 02720D6, 02720DZ, 02720E6, 0272346, 0272366, 0272376, 02723DZ, 027244Z, 027245Z, 02724D6, 02724EZ, 02724F6, 02724T6, 02724TZ, 02724ZZ, 0273046, 0273066, 027306Z, 0273076, 02730E6, 02730ZZ, 0273376, 02733DZ, 02733FZ, 02733GZ, 0273456, 0273466, 02734D6, 02734E6, 02734F6, 02734G6, 02734T6, 02C00Z6, 02C10ZZ, 02C20Z6, 021008C, 021009C, 021009W, 02100AF, 02100KF, 02100Z8, 02103D4, 0210444, 0210483, 0210493, 0210499, 02104D4, 02104JC, 02104K9, 02104Z3, 02104Z8, 02104ZF, 021108C, 0211093, 02110K3, 02110ZF, 0211483, 0211489, 02114A8, 02114AC, 02114JF, 0212089, 021208F, 02120AC, 02120AF, 02120AW, 02120J9, 02120JC, 021248C, 0212498, 02124AF, 02124J3, 02124J8, 02124K3, 02124ZC, 021308F, 021309C, 021309W, 02130A8, 02130AC, 02130J9, 02130Z8, 02130ZC, 02130ZF, 0213489, 02134A8, 02134AC, 02134J9, 02134Z8, 02134ZC, 02700DZ, 02700G6, 02700ZZ, 027034Z, 02703DZ, 02703FZ, 02704FZ, 02704ZZ, 027105Z, 02710F6, 027137Z, 02713D6, 02713E6, 0271446, 02714E6, 027204Z, 0272076, 02720F6, 02720T6, 02720Z6, 0272356, 027235Z, 02723F6, 02723T6, 02723ZZ, 0272476, 02724DZ, 02724E6, 0273056, 02730FZ, 02730TZ, 02733E6, 02733F6, 02733Z6, 027347Z, 02C00ZZ, 02C04Z6, 02C20ZZ, 02C23Z6, 02C23ZZ, 02C24Z6, 02C24ZZ, 02C30Z6, 02C33Z6, 02C33ZZ, 02C34ZZ, 3E07017, 3E070PZ, 0210083, 0210093, 02100A8, 02100AC, 02100J3, 02100JC, 02100JW, 02100K8, 02100KC, 02100Z3, 0210489, 021048W, 0210498, 02104A3, 02104A8, 02104J9, 02104JF, 02104JW, 02104K8, 02104KW, 0211083, 021108F, 021108W, 0211098, 021109C, 02110AC, 02110AW, 02110Z9, 021148C, 021149F, 02114A3, 02114AW, 02114J8, 02114JW, 02114Z9, 02114ZF, 0212098, 02120A8, 02120K9, 02120KF, 02120ZC, 02120ZF, 0212444, 0212483, 021248W, 0212493, 02124AC, 02124J9, 02124KC, 02124KF, 02124Z3, 0213089, 02130A9, 02130AW, 02130JC, 02130JF, 02130K8, 02130Z3, 0213344, 02133D4, 0213488, 021348F, 021349C, 02134A3, 02134J8, 02134K8, 02134K9, 02134ZF, 027005Z, 02700TZ, 0270366, 0270376, 02703T6, 0270456, 02704D6, 02704E6, 02704T6, 02704TZ, 0271056, 0271066, 02710Z6, 027134Z, 027135Z, 027136Z, 02713G6, 02713T6, 0271456, 027146Z, 02714G6, 02714ZZ, 02720G6, 02720ZZ, 027237Z, 027246Z, 02724G6, 027304Z, 027307Z, 02730T6, 027334Z, 027335Z, 0273366, 027336Z, 027337Z, 02733EZ, 02733TZ, 02734GZ, 02734Z6, 02C03Z6, 02C13Z6, 02C13ZZ, 02C34Z6, 3E073PZ, 0210088, 0210089, 021008W, 02100A3, 02100A9, 02100JF, 02100K3, 02100K9, 02100Z9, 02100ZF, 021048C, 021049F, 02104AC, 02104K3, 02104Z9, 02104ZC, 0211089, 0211099, 02110J8, 02110JC, 02110KC, 02110KW, 02110ZC, 0211344, 0211444, 0211488, 021148W, 0211493, 02114A9, 02114AF, 02114D4, 02114K8, 02114K9, 02114ZC, 0212083, 0212099, 021209F, 02120A3, 02120A9, 02120J3, 02120JF, 02120K3, 0212344, 0212489, 02124A3, 02124A9, 02124JF, 02124K9, 02124Z9, 021309F, 02130A3, 02130JW, 02130KC, 02130KW, 0213483, 021348C, 0213499, 021349F, 02134A9, 02134AF, 02134AW, 02134D4, 02134JC, 02134JW, 02134Z3, 02134Z9, 0270056, 0270076, 02700D6, 02700EZ, 02700F6, 02700Z6, 027037Z, 02703E6, 02703EZ, 0270446, 027044Z, 02704EZ, 02704G6, 02704Z6, 027106Z, 0271076, 027107Z, 02710EZ, 02710GZ, 02710TZ, 0271366, 0271376, 02713DZ, 02713EZ, 02713TZ, 027144Z, 027147Z, 02714D6, 02714F6, 02714T6, 02714Z6, 027207Z, 02720EZ, 027236Z, 02723D6, 02723FZ, 02723GZ, 02723TZ, 0272446, 0272456, 0272466, 02724FZ, 027305Z, 02730F6, 02730GZ, 02730Z6, 0273346, 0273356, 02733G6, 02733T6, 02733ZZ, 027345Z, 027346Z, 0273476, 02734DZ, 02734TZ, 02C30ZZ, 0210099, 02100J9, 02100KW, 02100ZC, 0210488, 021048F, 021049C, 02104A9, 02104AF, 02104AW, 02104KC, 02104KF, 021109W, 02110A3, 02110A8, 02110AF, 02110J3, 02110J9, 02110JW, 02110KF, 02110Z8, 021148F, 0211498, 021149C, 02114J3, 02114KC, 02114Z3, 021208C, 021208W, 0212093, 02120J8, 02120KC, 02120Z3, 02120Z9, 02123D4, 0212488, 0212499, 02124A8, 02124AW, 02124D4, 02124ZF, 0213083, 0213099, 02130AF, 02130J8, 02130K3, 02130K9, 02130Z9, 021348W, 0213498, 021349W, 02134J3, 02134K3, 0270046, 027004Z, 0270066, 02700FZ, 02700GZ, 02700T6, 027035Z, 027036Z, 02703G6, 02703ZZ, 0270466, 027046Z, 0270476, 02704F6, 02704GZ, 0271046, 027104Z, 02710D6, 02710DZ, 02710T6, 02710ZZ, 0271356, 02713F6, 02713GZ, 0271466, 0271476, 02714GZ, 0272046, 027205Z, 027206Z, 02720FZ, 02720GZ, 02720TZ, 027234Z, 02723E6, 02723EZ, 02723G6, 02723Z6, 027247Z, 02724GZ, 02724Z6, 02730D6, 02730DZ, 02730EZ, 02730G6, 02733D6, 0273446, 027344Z, 02734EZ, 02734FZ, 02734ZZ, 02C03ZZ, 02C04ZZ, 02C10Z6, 02C14Z6, 02C14ZZ, 3E07317  CPT/HCPCS: 33510, 33511, 33512, 33513, 33514, 33516, 33517, 33518, 33519, 33521, 33522, 33523, 33524, 33525, 33526, 33527, 33528, 33529, 33531, 33532, 33533, 33534, 33535, 33536, 92920, 92921, 92924, 92925, 92928, 92929, 92933, 92934, 92937, 92938, 92941, 92943, 92944, 92980, 92981, 92982, 92984, 92995, 92996, S2205, S2206, S2207, S2208, S2209, G0290, G0291 | IP, OP, ED, or GL | Claims or EHR |  |
| …Prior arrhythmias, dysrhythmias, or abnormal heartbeat | ICD-10-CM: I48.0, I48.1, I48.2, I48.3, I48.4, I48.91, I48.92 | IP, OP, ED, or GL | Claims or EHR | [Sentinel Initiative](https://www.sentinelinitiative.org/about/how-sentinel-gets-its-data),  [Fung, K. W., et al. (2016)](https://www.ncbi.nlm.nih.gov/pmc/articles/PMC4862764/) |
| …Hypertension | ICD-10-CM: I10, I16.9, I11.9, I11.0, I12.9, ICD-10-CM I12.0, I13.10, I13.0, I13.11, I13.2, I15.0, I15.8, I15.1, I15.2, I15.9, I16.0, I16.1 | IP, OP, ED, GL | Claims or EHR | [Sentinel Initiative](https://www.sentinelinitiative.org/about/how-sentinel-gets-its-data),  [Fung, K. W., et al. (2016)](https://www.ncbi.nlm.nih.gov/pmc/articles/PMC4862764/) |
| Diabetes | \| E11.9 \| Type 2 diabetes mellitus without complications \| ICD-10-CM \| \| --- \| --- \| --- \| \| E10.9 \| Type 1 diabetes mellitus without complications \| ICD-10-CM \| \| E11.65 \| Type 2 diabetes mellitus with hyperglycemia \| ICD-10-CM \| \| E10.65 \| Type 1 diabetes mellitus with hyperglycemia \| ICD-10-CM \| \| E11.69 \| Type 2 diabetes mellitus with other specified complication \| ICD-10-CM \| \| E13.10 \| Other specified diabetes mellitus with ketoacidosis without coma \| ICD-10-CM \| \| E10.10 \| Type 1 diabetes mellitus with ketoacidosis without coma \| ICD-10-CM \| \| E11.00 \| Type 2 diabetes mellitus with hyperosmolarity without nonketotic hyperglycemic-hyperosmolar coma (NKHHC) \| ICD-10-CM \| \| E11.01 \| Type 2 diabetes mellitus with hyperosmolarity with coma \| ICD-10-CM \| \| E10.69 \| Type 1 diabetes mellitus with other specified complication \| ICD-10-CM \| \| E11.641 \| Type 2 diabetes mellitus with hypoglycemia with coma \| ICD-10-CM \| \| E10.11 \| Type 1 diabetes mellitus with ketoacidosis with coma \| ICD-10-CM \| \| E10.641 \| Type 1 diabetes mellitus with hypoglycemia with coma \| ICD-10-CM \| \| E11.29 \| Type 2 diabetes mellitus with another diabetic kidney complication \| ICD-10-CM \| \| E10.29 \| Type 1 diabetes mellitus with another diabetic kidney complication \| ICD-10-CM \| \| E11.21 \| Type 2 diabetes mellitus with diabetic nephropathy \| ICD-10-CM \| \| E10.21 \| Type 1 diabetes mellitus with diabetic nephropathy \| ICD-10-CM \| \| E11.311 \| Type 2 diabetes mellitus with unspecified diabetic retinopathy with macular edema \| ICD-10-CM \| \| E11.319 \| Type 2 diabetes mellitus with unspecified diabetic retinopathy without macular edema \| ICD-10-CM \| \| E11.36 \| Type 2 diabetes mellitus with diabetic cataract \| ICD-10-CM \| \| E11.39 \| Type 2 diabetes mellitus with other diabetic ophthalmic complication \| ICD-10-CM \| \| E10.311 \| Type 1 diabetes mellitus with unspecified diabetic retinopathy with macular edema \| ICD-10-CM \| \| E10.319 \| Type 1 diabetes mellitus with unspecified diabetic retinopathy without macular edema \| ICD-10-CM \| \| E10.36 \| Type 1 diabetes mellitus with diabetic cataract \| ICD-10-CM \| \| E10.37X1 \| Type 1 diabetes mellitus with diabetic macular edema, resolved following treatment, right eye \| ICD-10-CM \| \| E10.37X2 \| Type 1 diabetes mellitus with diabetic macular edema, resolved following treatment, left eye \| ICD-10-CM \| \| E10.37X3 \| Type 1 diabetes mellitus with diabetic macular edema, resolved following treatment, bilateral \| ICD-10-CM \| | IP, OP, ED, or GL | Claims or EHR | [Sentinel Initiative](https://www.sentinelinitiative.org/),  [Fung, K. W., et al. (2016)](https://www.ncbi.nlm.nih.gov/pmc/articles/PMC4862764/) |
| History of smoking/tobacco use | ICD-10-CM: F17.21*, F17.22*, F17.29*, Z71.6, Z72.0, Z87.891, 099.33*, T65.21*  CPT: 99406, 99407, G0436, G0437, G9016, S9453, S4995, G9276, G9458, 1034F, 4004F, 4001F  NDC Generic Names: *nicotine*, *varenicline* | IP, OP, ED, or GL | Claims or EHR | Definition based in-part on [Desai et al, 2016](https://pubmed.ncbi.nlm.nih.gov/26764576/) and CMS General Equivalence Mappings. |
| History of alcohol use | ICD-10-CM: F10, E52, G62.1, I42.6, K29.2, K70.0, K70.3, K70.9, T51*, Z50.2, Z71.4, Z72.1 | IP, OP, ED, or GL | Claims or EHR | [Quan et al, 2005](https://pubmed.ncbi.nlm.nih.gov/16224307/) |
| Hyperlipidemia | ICD-10-CM: E78.0*-E78.6* | IP, OP, ED, or GL | Claims or EHR |  |
| …Immunosuppressive condition: Inflammatory bowel disease | ICD-10-CM: K50, K51 | IP, OP, ED, or GL | Claims or EHR |  |
| Immunosuppressive condition | NDC Generic Name: *ABATACEPT*, *ABATACEPT/MALTOSE*, *ADALIMUMAB*, *ANAKINRA*, *AZATHIOPRINE*, *AZATHIOPRINE SODIUM*, *BELATACEPT*, *BELIMUMAB*, *CERTOLIZUMAB PEGOL*, *CYCLOPHOSPHAMIDE*, *CYCLOSPORINE*, *CYCLOSPORINE, MODIFIED*, *EFALIZUMAB*, *ETANERCEPT*, *GOLIMUMAB*, *INFLIXIMAB*, *LEFLUNOMIDE*, *METHOTREXATE*, *METHOTREXATE SODIUM*, *METHOTREXATE SODIUM/PF*, *METHOTREXATE/PF*, *MYCOPHENOLATE MOFETIL*, *MYCOPHENOLATE MOFETIL HCL*, *MYCOPHENOLATE SODIUM*, *RITUXIMAB*, *SIROLIMUS*, *TOCILIZUMAB* | IP, OP, ED, or GL | Claims or EHR |  |
| Kidney Disease | ICD-10-CM: I12.0, I13.1, N03.2*-N03.7*, N05.2*– N05.7*, N17*, N18*, N19*, N25.0, Z49.0*– Z49.2*, Z94.0*, Z99.2 | IP, OP, ED, or GL | Claims or EHR | [Quan et al, 2005](https://pubmed.ncbi.nlm.nih.gov/16224307/) |
| Liver disease | ICD-10-CM: I85.0, I85.00, I85.01, I86.4, K70.4, K70.40, K70.41, K71.1, K71.10, K71.11, K72.1, K72.10, K72.11, K72.9, K72.90, K72.91, K76.5, K76.6, K76.7, B18, B18.0, B18.1, B18.2, B18.8, B18.9, K70.0, K70.1, K70.10, K70.11, K70.2, K70.3, K70.30, K70.31, K70.9, K71.3, K71.4, K71.5, K71.50, K71.51, K71.7, K73, K73.0, K73.1, K73.2, K73.8, K73.9, K74, K74.0, K74.1, K74.2, K74.3, K74.4, K74.5, K74.6, K74.60, K74.69, K76.0, K76.2, K76.3, K76.4, K76.8, K76.81, K76.89, K76.9, Z94.4 | IP, OP, ED, or GL | Claims or EHR |  |
| Overweight/obesity | ICD-10-CM: E66.9, Z68.1 | IP, OP, ED, or GL | Claims or EHR | [Ammann et al., 2018](https://pubmed.ncbi.nlm.nih.gov/30003617/) |
| **Medications In Baseline** |  |  |  |  |
| Angiotensin converting enzyme inhibitor, ACEi | NDC Generic Name: *fosinopril*, *moexipril*, *enalapril*, *perindopril*, *captopril*, *trandolapril*, *quinapril*, *ramipril*, *benazepril*, *lisinopril*  Brand Name: *Monopril*, *Univasc*, *Vasotec*, *Aceon*, *Capoten*, *Mavik*, *Accupril*, *Altace*, *Lotensin*, *Prinivil*, *Zestril* | IP, OP, ED, or GL | Claims or EHR |  |
| Angiotensin receptor blocker, ARB | NDC Generic Name: *azilsartan*, *eprosartan*, *candesartan*, *telmisartan*, *olmesartan*, *irbesartan*, *valsartan*, *losartan*  Brand Name: *Edarbi*, *Teveten*, *Atacand*, *Micardis*, *Benicar*, *Avapro*, *Diovan*, *Cozaar*, *Exforge*, *Avalide*, *Hyzaar* | IP, OP, ED, or GL | Claims or EHR |  |
| Antiarrhythmic medication | NDC Generic Name: *quinidine*, *disopyramide*, *propafenone*, *flecainide*, *phenytoin*, *mexiletine*, *lidocaine*, *procainamide*, *tocainide*, *acebutolol*, *esmolol*, *propranolol*, *ibutilide*, *dofetilide*, *sotalol*, *dronedarone*, *amiodarone*, *bretylium*, *verapamil*, *diltiazem*, *adenosine*, *digoxin*  Brand Name: *Adenocard*, *Adenoscan*, *Betapace*, *Brevibloc*, *Calan*, *Cardioquin*, *Cardizem*, *Cartia XT*, *Cordarone*, *Corvert*, *Covera-HS*, *DentiPatch*, *Digitek*, *Dilantin*, *Dilt-XR*, *Diltia XT*, *Hemangeol*, *Inderal*, *InnoPran XL*, *Isoptin SR*, *Lanoxicaps*, *Lanoxin*, *Mexitil*, *Multaq*, *Nexterone*, *Norpace*, *Pacerone*, *Phenytek*, *Phenytoin Sodium*, *Procanbid*, *Pronestyl*, *Quin-G*, *Quinidex Extentabs*, *Rythmol*, *Sectral*, *Sorine*, *Sotylize*, *Tambocor*, *Taztia XT*, *Tiazac*, *Tikosyn*, *Tonocard*, *UAD Caine*, *Verelan*, *Xylocaine HCl* | IP, OP, ED, or GL | Claims or EHR |  |
| Anticoagulant | NDC Generic Name: *apixaban*, *argatroban*, *desirudin*, *lepirudin*, *dabigatran*, *danaparoid*, *edoxaban*, *tinzaparin*, *heparin*, *ardeparin*, *bivalirudin*  Brand Name: *Eliquis*, *Acova*, *Iprivask*, *Refludan*, *Pradaxa*, *Orgaran*, *Savaysa*, *Innohep*, *Normiflo*, *Angiomax* | IP, OP, ED, or GL | Claims or EHR |  |
| Antifibrinolytics | NDC Generic Name: *abciximab*, *alteplase*, *amediplase*, *anagrelide*, *ancrod*, *anistreplase*, *argatroban*, *astaxanthin*, *brinase*, *dalteparin*, *danaparoid*, *defibrotide*, *desmoteplase*, *dicoumarol*, *drotrecogin alfa*, *edoxaban*, *ferric chloride*, *fibrinolysin*, *fondaparinux*, *iloprost*, *monteplase*, protein c, *reteplase*, *saruplase*, *streptokinase*, *sulodexide*, *tenecteplase*, *tinzaparin*, *urokinase* | IP, OP, ED, or GL | Claims or EHR |  |
| Antihypertensive | NDC Generic Name:, *fosinopril*, *moexipril*, *enalapril*, *perindopril*, *captopril*, *trandolapril*, *quinapril*, *ramipril*, *benazepril*, *lisinopril*, *azilsartan*, *eprosartan*, *candesartan*, *telmisartan*, *olmesartan*, *irbesartan*, *valsartan*, *losartan*, *prazosin*, *doxazosin*, *terazosin*, *acebutolol*, *atenolol*, *betaxolol*, *bisoprolol*, *carteolol*, *carvedilol*, *labetalol*, *metoprolol*, *nadolol*, *nebivolol*, *penbutolol*, *pindolol*, *propranolol*, *sotalol*, *timolol*, *esmolol*, *nitroglycerin*, *amlodipine*, *bepridil*, *clevidipine*, *diltiazem*, *felodipine*, *isradipine*, *nicardipine*, *nifedipine*, *nimodipine*, *nisoldipine*, *verapamil*, *diltiazem*, *pitavastatin*, *fluvastatin*, *rosuvastatin*, *pravastatin*, *lovastatin*, *atorvastatin*, *simvastatin*, *niacin*, *cholestyramine*, *colesevelam*, *colestipol*, *ezetimibe*, *fenofibrate*, *gemfibrozil*, *alirocumab*, *evolocumab*, *chlorothiazide*, *chlorthalidone*, *hydrochlorothiazide*, *indapamide*, *metolazone*, *bumetanide*, *ethacrynic acid*, *furosemide*, *torsemide*, *amiloride*, *epelrenone*, *spironolactone*, *triamterene*  Brand Name: *Monopril*, *Univasc*, *Vasotec*, *Aceon*, *Capoten*, *Mavik*, *Accupril*, *Altace*, *Lotensin*, *Prinivil*, *Zestril*, *Edarbi*, *Teveten*, *Atacand*, *Micardis*, *Benicar*, *Avapro*, *Diovan*, *Cozaar*, *Exforge*, *Avalide*, *Hyzaar*, *Minipress*, *Cardura*, *Hytrin*, *Sectral*, *Tenormin*, *Kerlone*, *Zebeta*, *Ziac*, *Cartrol*, *Coreg*, *Normodyne*, *Trandate*, *Lopressor*, *Toprol XL*, *Corgard*, *Bystolic*, *Levatol*, *Visken*, *Inderal*, *Innopran XL*, *Hemangeol*, *Betapace*, *Sorine*, *Sotylize*, *Blocadren*, *Brevibloc*, *Nitrolingual*, *Nitrostat*, *Nitromist*, *Nitro-bid*, *Nitro-dur*, *Transderm-nitro*, *Nitro-time*, *Isordil*, *Dilatrate SR*, *Norvasc*, *Caduet*, *Lotrel*, *Exforge*, *Twynsta*, *Azor*, *Tribenzor*, *Prestalia*, *Vascor*, *Cleviprex*, *Cardizem*, *Plendil*, *Dynacirc*, *Cardene*, *Adalat*, *Procardia*, *Sular*, *Calan*, *Isoptin*, *Verelan*, *Covera hs*, *Cardizem* | IP, OP, ED, or GL | Claims or EHR |  |
| Antibiotic agent (other than Azithromycin) | NDC Generic Name: *amikacin*, *gentamicin*, *kanamycin*, *neomycin*, *netilmicin*, *tobramycin*, *paromomycin*, *streptomycin*, *spectinomycin*, *plazomicin*, *geldanamycin*, *herbimycin*, *rifaximin*, *ertapenem*, *doripenem*, *cilastatin / imipenem*, *meropenem*, *cefadroxil*, *cefazolin*, *cephradine*, *cephapirin*, *cephalothin*, *cefalexin*, *cephalexin*, *cefaclor*, *cefoxitin*, *cefotetan*, *cefamandole*, *cefmetazole*, *cefonicid*, *loracarbef*, *cefprozil*, *cefuroxime*, *cefixime*, *cefdinir*, *cefditoren*, *cefoperazone*, *cefotaxime*, *cefpodoxime*, *ceftazidime*, *ceftibuten*, *ceftizoxime*, *moxalactam*, *ceftriaxone*, *cefepime*, *cefiderocol*, *ceftaroline*, *ceftobiprole*, *teicoplanin*, *vancomycin*, *telavancin*, *dalbavancin*, *oritavancin*, *clindamycin*, *lincomycin*, *daptomycin*, *clarithromycin*, *erythromycin*, *roxithromycin*, *telithromycin*, *spiramycin*, *fidaxomicin*, *aztreonam*, *furazolidone*, *nitrofurantoin*, *linezolid*, *tedizolid*, *posizolid*, *radezolid*, *amoxicillin*, *ampicillin*, *azlocillin*, *dicloxacillin*, *flucloxacillin*, *mezlocillin*, *carbenicillin*, *methicillin*, *nafcillin*, *oxacillin*, *penicillin v*, *piperacillin*, *penicillin g*, *temocillin*, *ticarcillin*, *amoxicillin/clavulanate*, *ampicillin/sulbactam*, *piperacillin/tazobactam*, *ticarcillin/clavulanate*, *bacitracin*, *colistin*, *polymyxin b*, *ciprofloxacin*, *enoxacin*, *gatifloxacin*, *gemifloxacin*, *levofloxacin*, *lomefloxacin*, *moxifloxacin*, *nadifloxacin*, *nalidixic acid*, *norfloxacin*, *ofloxacin*, *delafloxacin*, *besifloxacin*, *cinoxacin*, *ciprofloxacin*, *trovafloxacin*, *grepafloxacin*, *sparfloxacin*, *temafloxacin*, *mafenide*, *sulfacetamide*, *sulfadiazine*, *silver sulfadiazine*, *sulfadimethoxine*, *sulfamethizole*, *sulfamethoxazole*, *sulfasalazine*, *sulfisoxazole*, *sulfamethoxazole/trimethoprim*, *sulfonamidochrysoidine*, *demeclocycline*, *doxycycline*, *metacycline*, *minocycline*, *oxytetracycline*, *tetracycline*, *sarecycline*, *omadacycline*, *eravacycline*, *clofazimine*, *thalidomide*, *dapsone*, *capreomycin*, *cycloserine*, *ethambutol*, *ethionamide*, *isoniazid*, *pyrazinamide*, *rifampin*, *rifamycin*, *rifabutin*, *rifapentine*, *streptomycin*, *arsphenamine*, *chloramphenicol(bs)*, *fosfomycin*, *fusidic acid*, *metronidazole*, *mupirocin*, *platensimycin*, *quinupristin/dalfopristin*, *thiamphenicol*, *tigecycline*, *tinidazole*, *trimethoprim*, *aztreonam*, *lefamulin*, *secnidazole*, *atovaquone*, *colistimethate*, *erythromycin / sulfisoxazole*, *trimetrexate*, *pentamidine*, *amoxicillin/clarithromycin/lansoprazole*, *vidarabine*, *ganciclovir*, *natamycin*, *trifluridine*  Brand Name: *Amikin*, *Amiglyde-V*, *Arikayce*, *Garamycin*, *Kantrex*, *Neo-Fradin*, *Neo-Tab*, *Neosporin*, *Netromycin*, *Nebcin*, *Tobradex*, *Tobi*, *Bethkis*, *Kitabis Pak*, *Tobrasol*, *Tomycine*, *Tobrex*, *Humatin*, *Trobicin*, *Zemdri*, *Xifaxan*, *Invanz*, *Doribax*, *Primaxin*, *Merrem*, *Duricef*, *Ancef*, *Kefzol*, *Velosef*, *Keflex*, *Biocef*, *Panixine*, *Distaclor*, *Ceclor*, *Raniclor*, *Mefoxin*, *Cefotan*, *Lorabid*, *Cefzil*, *Ceftin*, *Zinacef*, *Suprax*, *Omnicef*, *Cefdiel*, *Spectracef*, *Meiact*, *Cefobid*, *Claforan*, *Vantin*, *Banadoz*, *Fortaz*, *Ceptaz*, *Tazicef*, *Cedax*, *Cefizox*, *Latamoxef*, *Rocephin*, *Maxipime*, *Fetroja*, *Teflaro*, *Zeftera*, *Targocid*, *Vancocin*, *Firvanq*, *Lyphocin*, *Vibativ*, *Dalvance*, *Orbactiv*, *Cleocin*, *Lincocin*, *Cubicin*, *Biaxin*, *Erythrocin*, *Erythroped*, *Ery-Tab*, *Eryc*, *EryPed*, *Ilosone*, *Ketek*, *Rovamycine*, *Dificid*, *Azactam*, *Furoxone*, *Macrodantin*, *Macrobid*, *Furadantin*, *Zyvox*, *Sivextro*, *Moxatag*, *Amoxil*, *Larotid*, *Trimox*, *Moxilin*, *Amoxicot*, *Apo-Amoxi*, *DisperMox*, *Principen*, *Ampi*, *Omnipen*, *Penglobe*, *Totacillin*, *Dycill*, *Dynapen*, *Pathocil*, *Floxapen*, *Mezlin*, *Geocillin*, *Staphcillin*, *Unipen*, *Nafcil*, *Nallpen*, *Bactocill*, *Veetids*, *Pen-Vee-K*, *Penicillin VK*, *PC Pen VK*, *Pipracil*, *Pfizerpen*, *Negaban*, *Ticar*, *Augmentin*, *Unasyn*, *Zosyn*, *Timentin*, *BACiiM*, *Baciguent*, *Coly-Mycin-S*, *Cipro*, *Ciproxin*, *Ciprobay*, *Ciloxan*, *Penetrex*, *Tequin*, *Zymar*, *Zymaxid*, *Factive*, *NegGram*, *Levaquin*, *Quixin*, *Iquix*, *Maxaquin*, *Avelox*, *Vigamox*, *Moxeza*, *NegGram*, *Noroxin*, *Floxin*, *Ocuflox*, *Baxdela*, *Besivance*, *Cinobac*, *Cipro*, *Proquin XR*, *Trovan*, *Raxar*, *Zagam*, *Omniflox*, *Sulfamylon*, *Sulamyd*, *Bleph-10*, *Cetamide*, *Microsulfon*, *Silvadene*, *Di-Methox*, * Albon*, *Thiosulfil Forte*, *Gantanol*, *Azulfidine*, *Gantrisin*, *Trixazole*, *Bactrim*, *Septra*, *Sulfatrim*, *Prontosil*, *Declomycin*, *Vibramycin*, *Adoxa*, *Oraxyl*, *Oracea*, *Monodox*, *Doxy*, *Doryx*, *Alodox*, *Avidoxy*, *Morgidox*, *NutriDox*, *Ocudox*, *Periostat*, *Minocin*, *Dynacin*, *Solodyn*, *Ximino*, *Minolira*, *Myrac*, *Terramycin*, *Sumycin*, *Achromycin V*, *Steclin*, *Actisite*, *Ala-Tet*, *Brodspec*, *Panmycin*, *Tetracap*, *Tetracon*, *Seysara*, *Nuzyra*, *Xerava*, *Lamprene*, *Thalomid*, *Avlosulfon*, *Aczone*, *Capastat*, *Seromycin*, *Myambutol*, *Trecator*, *Hydra*, *Hyzyd*, *Isovit*, *Nydrazid*, *Aldinamide*, *Rifadin*, *Rimactane*, *Aemcolo*, *Mycobutin*, *Priftin*, *Salvarsan*, *Chloromycetin*, *Monurol*, *Monuril*, *Fucidin*, *Flagyl*, *Bactroban*, *Synercid*, *Tygacil*, *Tindamax Fasigyn*, *Proloprim*, *Trimpex*, *Cayston*, *Azactam*, *Xenleta*, *Solosec*, *Mepron*, *Coly Mycin M*, *Eryzole*, *Pediazole*, *Neutrexin*, *Pentam*, *Omeclamox-PAK*, *Prevpac*, *Vira-A*, *Vitrasert*, *Zigran*, *Natacyn*, *Viroptic* | IP, OP, ED, or GL | Claims or EHR |  |
| Antiplatelet medication | NDC Generic Name: *prasugrel*, *ticagrelor*, *clopidogrel*, *aspirin*, *aspirin/dipyridamole*, *vorapaxar*, *cilostazol*, *dipyridamole*, *ticlopidine*, *eptifibatide*, *abciximab*, *aspirin/omeprazole*, *cangrelor*, *ticagrelor*, *tirofiban*, *triflusal*  Brand Name: *Effient*, *Brilinta*, *Plavix*, *Aspirin*, *Asaphen*, *Entrophen*, *Novasen*, *Aspro clear*, *Disprin*, *Zorprin*, *Durlaza*, *Asatab*, *Adprin-B*, *Arthritis pain formula*, *Ascriptin*, *Bufferin*, *Ecotrin*, *Empirin*, *Halfprin*, *Asa*, *Aggrenox*, *Zontivity*, *Pletaal*, *Persantine*, *Ticlid*, *Integrilin*, *Reopro*, *Yosprala*, *Kengreal*, *Brilinta*, *Aggrastat*, *Disgren* | IP, OP, ED, or GL | Claims or EHR |  |
| Antivirals (other than HCQ, Remdesivir) | NDC Generic Name: *abacavir*, *acyclovir *, *aciclovir*, *adefovir*, *amantadine*, *amprenavir*, *atazanavir*, *baloxavir marboxil *, *bictegravir*, *boceprevir*, *cidofovir*, *cobicistat *, *daclatasvir*, *darunavir*, *delavirdine*, *danoprevir*, *didanosine*, *docosanol*, *dolutegravir*, *doravirine*, *edoxudine*, *efavirenz*, *elvitegravir*, *emtricitabine*, *emtricitabine/tenofovir*, *enfuvirtide*, *entecavir*, *etravirine*, *famciclovir*, *favipiravir*, *fomivirsen*, *fosamprenavir*, *foscarnet*, *fosfonat*, *ganciclovir *, *galidesivir*, *ibalizumab *, *idoxuridine*, *imiquimod*, *inosine pranobex*, *indinavir*, *interferon alfa*, *interferon beta*, *interferon*, *lamivudine*, *letermovir*, *lopinavir*, *maraviroc*, *methisazone*, *moroxydine*, *nelfinavir*, *nevirapine*, *nexavir*, *nitazoxanide*, *oseltamivir*, *peginterferon alfa-2a*, *peginterferon alfa-2b*, *penciclovir*, *peramivir *, *pleconaril*, *podophyllotoxin*, *raltegravir*, *rilpivirine *, *rimantadine*, *ritonavir*, *rintatolimod*, *saquinavir*, *simeprevir*, *sofosbuvir*, *stavudine*, *taribavirin*, *telaprevir*, *telbivudine*, *tenofovir alafenamide*, *tenofovir disoproxil*, *tenofovir*, *tipranavir*, *trifluridine*, *tromantadine*, *umifenovir *, *valaciclovir *, *valganciclovir*, *vicriviroc*, *vidarabine*, *viramidine*, *zalcitabine*, *zanamivir*, *zidovudine*, *Ziagen*, *Sitavig*  Brand Name: *Zovirax*, *Preveon*, *Hepsera*, *Gocovri*, *Agenerase*, *Reyataz*, *Xofluza*, *Biktarvy*, *Victrelis*, *Visitide*, *Tybost*, *Daklinza*, *Prezista*, *Rescriptor*, *Ganovo*, *Videx*, *Abreva*, *Dovato*, *Tivicay*, *Pifeltro*, *Sustiva*, *Symfi*, *Vitekta*, *Descovy*, *Truvada*, *Atripla*, *Biktarvy*, *Fuzeon*, *Baraclude*, *Intelence*, *Famvir*, *Avigan*, *Vitravene*, *Lexiva*, *Foscavir*, *Cytovene*, *Trogarzo*, *Dendrid*, *Herplex*, *Zyclara*, *Aldara*, *Imunovir*, *Crixivan*, *Intron A*, *Rebif*, *Roferon-A*, *Betaseron*, *Extavia*, *Avonex*, *Interfergen*, *Plegridy*, *Actimmune*, *Alferon*, *Combivir*, *Prevymis*, *Kaletra*, *Selzentry*, *Celsentri*, *Tapazole*, *Viracept*, *Viramune*, *Kutapressin*, *Alinia*, *Tamiflu*, *Pegasys*, *PegIntron*, *Pegridy*, *Denavir*, *Rapivab*, *Picovir*, *Condylox*, *Isentress*, *Edurant*, *Rebetol*, *Flumadine*, *Novir*, *Ampligen*, *Invirase*, *Fortovase*, *Olysio*, *Sovaldi*, *Zerit*, *Incivek*, *Incivo*, *Tyzeka*, *Odefsey*, *Complera*, *Vemlidy*, *Viread*, *Aptivus*, *Viroptic*, *Viru-Merz*, *Arbidol*, *Valtrex*, *Valcyte*, *Vira-A*, *Hivid*, *Relenza*, *Retrovir*, *Epzicom*, *Stribild*, *Zepatier*, *Dovato*, *Trizivir*, *Mavyret*, *Harvoni*, *Triumeq*, *Epclusa*, *Symtuza*, *Genvoya*, *Viekira*, *Juluca*, *Combivir*, *Prezcobix*, *AccessPak*, *Cimduo*, *Delstrigo*, *Evotaz*, *Technivie*, *Temixys*, *Vosevi* | IP, OP, ED, or GL | Claims or EHR |  |
| Famotidine | NDC Generic Name: *famotidine*  Brand Name: TBD | IP, OP, ED, or GL | Claims or EHR |  |
| Novel oral anticoagulant (NOAC) | NDC Generic Name: *apixaban*, *dabigatran*, *rivaroxaban*, *edoxaban*  Brand Name: *Eliquis*, *Pradaxa*, *Xarelto*, *Savaysa* | IP, OP, ED, or GL | Claims or EHR |  |
| Oral anti-diabetic medication | NDC Generic Name: *metformin*, *canagliflozin*, *dapagliflozin*, *empagliflozin*, *ertugliflozin*, *alogliptin*, *linagliptin*, *miglitol*, *pioglitazone*, *rosiglitazone*, *saxagliptin*, *sitagliptin*, *nateglinide*, *glipizide*, *repaglinide*, *glyburide*, *glibenclamide*, *glimepiride*, *tolbutamide*, *tolazamide*, *chlorpropamide*, *gliclazide*, *acarbose*, *voglibose*, *vildagliptin*, *bromocriptine*, *teneligliptin*, *trelagliptin*, *anagliptin*  Brand Name: , *Glucophage*, *Glumetza*, *Riomet*, *Fortamet*, *Invokana*, *Invokamet*, *Farxiga*, *Xigduo xr*, *Qtern*, *Jardiance*, *Nesina*, *Tradjenta*, *Glyset*, *Actos*, *Avandia*, *Onglyza*, *Januvia*, *Starlix*, *Glucotrol*, *Prandin*, *Glynase*, *Micronase*, *Glycron*, *Dibeta*, *Amaryl*, *Tol-tab*, *Tolinase*, *Diabinese*, *Diamicron*, *Precose*, *Voglib*, *Galvus*, *Parlodel*, *Cycloset*, *Tenelia*, *Zafatek*, *Suiny*, *Qternmet xr*, *Trijardy xr*, *Synjardy*, *Segluromet*, *Metaglip*, *Glucovance*, *Jentadueto*, *Actoplus met*, *Prandimet*, *Avandamet*, *Kombiglyze*, *Janumet*, *Kazano*, *Steglatro*, *Rybelsus* | IP, OP, ED, or GL | Claims or EHR |  |
| Remdesivir | NDC Generic Name: *remdesivir* | IP, OP, ED, or GL | Claims or EHR |  |
| Statin | NDC Generic Name: *pitavastatin*, *fluvastatin*, *rosuvastatin*, *pravastatin*, *lovastatin*, *atorvastatin*, *simvastatin*  Brand Name: *Livalo*, *Lescol*, *Crestor*, *Pravachol*, *Mevacor*, *Altoprev*, *Lipitor*, *Zocor* | IP, OP, ED, or GL | Claims or EHR |  |
| Systemic glucocorticoid | NDC Generic Name: *prednisone*, *prednisolone*, *methylprednisolone*, *hydrocortisone*, *betamethasone*,*triamcinolone*,*dexamethasone*  CPT/HCPCS codes: J2650, J2920, J7506, J1690, J2640, J1720, J2930, J1020, J1030, J1040, J1700, J1710, J7512, 80420, C9034, C9256, J1094, J1095, J1100, J7312, J7637, J7638, J8540, K0512, K0513, S0173 | IP, OP, ED, or GL | Claims or EHR |  |
| Tocilizumab | NDC Generic Name: *tocilizumab*  Brand Name: *Actemra*  CPT/HCPCS codes: C9264, J3262 | IP, OP, ED, or GL | Claims or EHR |  |

1. Unless otherwise noted, variables in this table are assessed in the 365 days prior to the index date.
2. IP=Inpatient; OP=Outpatient; ED=Emergency Department; GL=Group Living Facility

Table 2.1-Immunosuppressive condition (inflammatory bowel disease, rheumatological diseases, and psoriasis)^1^

| Class | Subclass | Code | Type | Description |
| --- | --- | --- | --- | --- |
| IBD | Crohn’s | 5550 | ICD-9-CM | Regional enteritis of small intestine |
| IBD | Crohn’s | 5551 | ICD-9-CM | Regional enteritis of large intestine |
| IBD | Crohn’s | 5552 | ICD-9-CM | Regional enteritis of small intestine with large intestine |
| IBD | Crohn’s | 5559 | ICD-9-CM | Regional enteritis of unspecified site |
| IBD | Crohn’s | K5000 | ICD-10-CM | Crohn's disease of small intestine without complications |
| IBD | Crohn’s | K50011 | ICD-10-CM | Crohn's disease of small intestine with rectal bleeding |
| IBD | Crohn’s | K50012 | ICD-10-CM | Crohn's disease of small intestine with intestinal obstruction |
| IBD | Crohn’s | K50013 | ICD-10-CM | Crohn's disease of small intestine with fistula |
| IBD | Crohn’s | K50014 | ICD-10-CM | Crohn's disease of small intestine with abscess |
| IBD | Crohn’s | K50018 | ICD-10-CM | Crohn's disease of small intestine with other complication |
| IBD | Crohn’s | K50019 | ICD-10-CM | Crohn's disease of small intestine with unspecified complications |
| IBD | Crohn’s | K5010 | ICD-10-CM | Crohn's disease of large intestine without complications |
| IBD | Crohn’s | K50111 | ICD-10-CM | Crohn's disease of large intestine with rectal bleeding |
| IBD | Crohn’s | K50112 | ICD-10-CM | Crohn's disease of large intestine with intestinal obstruction |
| IBD | Crohn’s | K50113 | ICD-10-CM | Crohn's disease of large intestine with fistula |
| IBD | Crohn’s | K50114 | ICD-10-CM | Crohn's disease of large intestine with abscess |
| IBD | Crohn’s | K50118 | ICD-10-CM | Crohn's disease of large intestine with other complication |
| IBD | Crohn’s | K50119 | ICD-10-CM | Crohn's disease of large intestine with unspecified complications |
| IBD | Crohn’s | K5080 | ICD-10-CM | Crohn's disease of both small and large intestine without complications |
| IBD | Crohn’s | K50811 | ICD-10-CM | Crohn's disease of both small and large intestine with rectal bleeding |
| IBD | Crohn’s | K50812 | ICD-10-CM | Crohn's disease of both small and large intestine with intestinal obstruction |
| IBD | Crohn’s | K50813 | ICD-10-CM | Crohn's disease of both small and large intestine with fistula |
| IBD | Crohn’s | K50814 | ICD-10-CM | Crohn's disease of both small and large intestine with abscess |
| IBD | Crohn’s | K50818 | ICD-10-CM | Crohn's disease of both small and large intestine with other complication |
| IBD | Crohn’s | K50819 | ICD-10-CM | Crohn's disease of both small and large intestine with unspecified complications |
| IBD | Crohn’s | K5090 | ICD-10-CM | Crohn's disease, unspecified, without complications |
| IBD | Crohn’s | K50911 | ICD-10-CM | Crohn's disease, unspecified, with rectal bleeding |
| IBD | Crohn’s | K50912 | ICD-10-CM | Crohn's disease, unspecified, with intestinal obstruction |
| IBD | Crohn’s | K50913 | ICD-10-CM | Crohn's disease, unspecified, with fistula |
| IBD | Crohn’s | K50914 | ICD-10-CM | Crohn's disease, unspecified, with abscess |
| IBD | Crohn’s | K50918 | ICD-10-CM | Crohn's disease, unspecified, with other complication |
| IBD | Crohn’s | K50919 | ICD-10-CM | Crohn's disease, unspecified, with unspecified complications |
| IBD | UC | 5560 | ICD-9-CM | Ulcerative (chronic) enterocolitis |
| IBD | UC | 5561 | ICD-9-CM | Ulcerative (chronic) ileocolitis |
| IBD | UC | 5562 | ICD-9-CM | Ulcerative (chronic) proctitis |
| IBD | UC | 5563 | ICD-9-CM | Ulcerative (chronic) proctosigmoiditis |
| IBD | UC | 5564 | ICD-9-CM | Pseudopolyposis of colon |
| IBD | UC | 5565 | ICD-9-CM | Left-sided ulcerative (chronic) colitis |
| IBD | UC | 5566 | ICD-9-CM | Universal ulcerative (chronic) colitis |
| IBD | UC | 5568 | ICD-9-CM | Other ulcerative colitis |
| IBD | UC | 5569 | ICD-9-CM | Ulcerative colitis, unspecified |
| IBD | UC | K5100 | ICD-10-CM | Ulcerative (chronic) pancolitis without complications |
| IBD | UC | K51011 | ICD-10-CM | Ulcerative (chronic) pancolitis with rectal bleeding |
| IBD | UC | K51012 | ICD-10-CM | Ulcerative (chronic) pancolitis with intestinal obstruction |
| IBD | UC | K51013 | ICD-10-CM | Ulcerative (chronic) pancolitis with fistula |
| IBD | UC | K51014 | ICD-10-CM | Ulcerative (chronic) pancolitis with abscess |
| IBD | UC | K51018 | ICD-10-CM | Ulcerative (chronic) pancolitis with other complication |
| IBD | UC | K51019 | ICD-10-CM | Ulcerative (chronic) pancolitis with unspecified complications |
| IBD | UC | K5120 | ICD-10-CM | Ulcerative (chronic) proctitis without complications |
| IBD | UC | K51211 | ICD-10-CM | Ulcerative (chronic) proctitis with rectal bleeding |
| IBD | UC | K51212 | ICD-10-CM | Ulcerative (chronic) proctitis with intestinal obstruction |
| IBD | UC | K51213 | ICD-10-CM | Ulcerative (chronic) proctitis with fistula |
| IBD | UC | K51214 | ICD-10-CM | Ulcerative (chronic) proctitis with abscess |
| IBD | UC | K51218 | ICD-10-CM | Ulcerative (chronic) proctitis with other complication |
| IBD | UC | K51219 | ICD-10-CM | Ulcerative (chronic) proctitis with unspecified complications |
| IBD | UC | K5130 | ICD-10-CM | Ulcerative (chronic) rectosigmoiditis without complications |
| IBD | UC | K51311 | ICD-10-CM | Ulcerative (chronic) rectosigmoiditis with rectal bleeding |
| IBD | UC | K51312 | ICD-10-CM | Ulcerative (chronic) rectosigmoiditis with intestinal obstruction |
| IBD | UC | K51313 | ICD-10-CM | Ulcerative (chronic) rectosigmoiditis with fistula |
| IBD | UC | K51314 | ICD-10-CM | Ulcerative (chronic) rectosigmoiditis with abscess |
| IBD | UC | K51318 | ICD-10-CM | Ulcerative (chronic) rectosigmoiditis with other complication |
| IBD | UC | K51319 | ICD-10-CM | Ulcerative (chronic) rectosigmoiditis with unspecified complications |
| IBD | UC | K5140 | ICD-10-CM | Inflammatory polyps of colon without complications |
| IBD | UC | K51411 | ICD-10-CM | Inflammatory polyps of colon with rectal bleeding |
| IBD | UC | K51412 | ICD-10-CM | Inflammatory polyps of colon with intestinal obstruction |
| IBD | UC | K51413 | ICD-10-CM | Inflammatory polyps of colon with fistula |
| IBD | UC | K51414 | ICD-10-CM | Inflammatory polyps of colon with abscess |
| IBD | UC | K51418 | ICD-10-CM | Inflammatory polyps of colon with other complication |
| IBD | UC | K51419 | ICD-10-CM | Inflammatory polyps of colon with unspecified complications |
| IBD | UC | K5150 | ICD-10-CM | Left sided colitis without complications |
| IBD | UC | K51511 | ICD-10-CM | Left sided colitis with rectal bleeding |
| IBD | UC | K51512 | ICD-10-CM | Left sided colitis with intestinal obstruction |
| IBD | UC | K51513 | ICD-10-CM | Left sided colitis with fistula |
| IBD | UC | K51514 | ICD-10-CM | Left sided colitis with abscess |
| IBD | UC | K51518 | ICD-10-CM | Left sided colitis with other complication |
| IBD | UC | K51519 | ICD-10-CM | Left sided colitis with unspecified complications |
| IBD | UC | K5180 | ICD-10-CM | Other ulcerative colitis without complications |
| IBD | UC | K51811 | ICD-10-CM | Other ulcerative colitis with rectal bleeding |
| IBD | UC | K51812 | ICD-10-CM | Other ulcerative colitis with intestinal obstruction |
| IBD | UC | K51813 | ICD-10-CM | Other ulcerative colitis with fistula |
| IBD | UC | K51814 | ICD-10-CM | Other ulcerative colitis with abscess |
| IBD | UC | K51818 | ICD-10-CM | Other ulcerative colitis with other complication |
| IBD | UC | K51819 | ICD-10-CM | Other ulcerative colitis with unspecified complications |
| IBD | UC | K5190 | ICD-10-CM | Ulcerative colitis, unspecified, without complications |
| IBD | UC | K51911 | ICD-10-CM | Ulcerative colitis, unspecified with rectal bleeding |
| IBD | UC | K51912 | ICD-10-CM | Ulcerative colitis, unspecified with intestinal obstruction |
| IBD | UC | K51913 | ICD-10-CM | Ulcerative colitis, unspecified with fistula |
| IBD | UC | K51914 | ICD-10-CM | Ulcerative colitis, unspecified with abscess |
| IBD | UC | K51918 | ICD-10-CM | Ulcerative colitis, unspecified with other complication |
| IBD | UC | K51919 | ICD-10-CM | Ulcerative colitis, unspecified with unspecified complications |
| Psoriasis | PA | 6960 | ICD-9-CM | Psoriatic arthropathy |
| Psoriasis | PA | L4050 | ICD-10-CM | Arthropathic psoriasis, unspecified |
| Psoriasis | PA | L4051 | ICD-10-CM | Distal interphalangeal psoriatic arthropathy |
| Psoriasis | PA | L4052 | ICD-10-CM | Psoriatic arthritis mutilans |
| Psoriasis | PA | L4054 | ICD-10-CM | Psoriatic juvenile arthropathy |
| Psoriasis | PA | L4059 | ICD-10-CM | Other psoriatic arthropathy |
| Psoriasis | PP | 6961 | ICD-9-CM | Other psoriasis |
| Psoriasis | PP | 6968 | ICD-9-CM | Other psoriasis and similar disorders |
| Psoriasis | PP | L400 | ICD-10-CM | Psoriasis vulgaris |
| Psoriasis | PP | L401 | ICD-10-CM | Generalized pustular psoriasis |
| Psoriasis | PP | L404 | ICD-10-CM | Guttate psoriasis |
| Psoriasis | PP | L4053 | ICD-10-CM | Psoriatic spondylitis |
| Psoriasis | PP | L408 | ICD-10-CM | Other psoriasis |
| Psoriasis | PP | L409 | ICD-10-CM | Psoriasis, unspecified |
| Rheum | AS | 7200 | ICD-9-CM | Ankylosing spondylitis |
| Rheum | AS | M081 | ICD-10-CM | Juvenile ankylosing spondylitis |
| Rheum | AS | M450 | ICD-10-CM | Ankylosing spondylitis of multiple sites in spine |
| Rheum | AS | M451 | ICD-10-CM | Ankylosing spondylitis of occipito-atlanto-axial region |
| Rheum | AS | M452 | ICD-10-CM | Ankylosing spondylitis of cervical region |
| Rheum | AS | M453 | ICD-10-CM | Ankylosing spondylitis of cervicothoracic region |
| Rheum | AS | M454 | ICD-10-CM | Ankylosing spondylitis of thoracic region |
| Rheum | AS | M455 | ICD-10-CM | Ankylosing spondylitis of thoracolumbar region |
| Rheum | AS | M456 | ICD-10-CM | Ankylosing spondylitis lumbar region |
| Rheum | AS | M457 | ICD-10-CM | Ankylosing spondylitis of lumbosacral region |
| Rheum | AS | M458 | ICD-10-CM | Ankylosing spondylitis sacral and sacrococcygeal region |
| Rheum | AS | M459 | ICD-10-CM | Ankylosing spondylitis of unspecified sites in spine |
| Rheum | CAPS | 75989 | ICD-9-CM | Other specified congenital anomalies |
| Rheum | CAPS | M042 | ICD-10-CM | Cryopyrin-associated periodic syndromes |
| Rheum | JIA | 71430 | ICD-9-CM | Polyarticular juvenile rheumatoid arthritis, chronic or unspecified |
| Rheum | JIA | 71431 | ICD-9-CM | Polyarticular juvenile rheumatoid arthritis, acute |
| Rheum | JIA | 71432 | ICD-9-CM | Pauciarticular juvenile rheumatoid arthritis |
| Rheum | JIA | 71433 | ICD-9-CM | Monoarticular juvenile rheumatoid arthritis |
| Rheum | JIA | M0800 | ICD-10-CM | Unspecified juvenile rheumatoid arthritis of unspecified site |
| Rheum | JIA | M08011 | ICD-10-CM | Unspecified juvenile rheumatoid arthritis, right shoulder |
| Rheum | JIA | M08012 | ICD-10-CM | Unspecified juvenile rheumatoid arthritis, left shoulder |
| Rheum | JIA | M08019 | ICD-10-CM | Unspecified juvenile rheumatoid arthritis, unspecified shoulder |
| Rheum | JIA | M08021 | ICD-10-CM | Unspecified juvenile rheumatoid arthritis, right elbow |
| Rheum | JIA | M08022 | ICD-10-CM | Unspecified juvenile rheumatoid arthritis, left elbow |
| Rheum | JIA | M08029 | ICD-10-CM | Unspecified juvenile rheumatoid arthritis, unspecified elbow |
| Rheum | JIA | M08031 | ICD-10-CM | Unspecified juvenile rheumatoid arthritis, right wrist |
| Rheum | JIA | M08032 | ICD-10-CM | Unspecified juvenile rheumatoid arthritis, left wrist |
| Rheum | JIA | M08039 | ICD-10-CM | Unspecified juvenile rheumatoid arthritis, unspecified wrist |
| Rheum | JIA | M08041 | ICD-10-CM | Unspecified juvenile rheumatoid arthritis, right hand |
| Rheum | JIA | M08042 | ICD-10-CM | Unspecified juvenile rheumatoid arthritis, left hand |
| Rheum | JIA | M08049 | ICD-10-CM | Unspecified juvenile rheumatoid arthritis, unspecified hand |
| Rheum | JIA | M08051 | ICD-10-CM | Unspecified juvenile rheumatoid arthritis, right hip |
| Rheum | JIA | M08052 | ICD-10-CM | Unspecified juvenile rheumatoid arthritis, left hip |
| Rheum | JIA | M08059 | ICD-10-CM | Unspecified juvenile rheumatoid arthritis, unspecified hip |
| Rheum | JIA | M08061 | ICD-10-CM | Unspecified juvenile rheumatoid arthritis, right knee |
| Rheum | JIA | M08062 | ICD-10-CM | Unspecified juvenile rheumatoid arthritis, left knee |
| Rheum | JIA | M08069 | ICD-10-CM | Unspecified juvenile rheumatoid arthritis, unspecified knee |
| Rheum | JIA | M08071 | ICD-10-CM | Unspecified juvenile rheumatoid arthritis, right ankle and foot |
| Rheum | JIA | M08072 | ICD-10-CM | Unspecified juvenile rheumatoid arthritis, left ankle and foot |
| Rheum | JIA | M08079 | ICD-10-CM | Unspecified juvenile rheumatoid arthritis, unspecified ankle and foot |
| Rheum | JIA | M0808 | ICD-10-CM | Unspecified juvenile rheumatoid arthritis, vertebrae |
| Rheum | JIA | M0809 | ICD-10-CM | Unspecified juvenile rheumatoid arthritis, multiple sites |
| Rheum | JIA | M0820 | ICD-10-CM | Juvenile rheumatoid arthritis with systemic onset, unspecified site |
| Rheum | JIA | M08211 | ICD-10-CM | Juvenile rheumatoid arthritis with systemic onset, right shoulder |
| Rheum | JIA | M08212 | ICD-10-CM | Juvenile rheumatoid arthritis with systemic onset, left shoulder |
| Rheum | JIA | M08219 | ICD-10-CM | Juvenile rheumatoid arthritis with systemic onset, unspecified shoulder |
| Rheum | JIA | M08221 | ICD-10-CM | Juvenile rheumatoid arthritis with systemic onset, right elbow |
| Rheum | JIA | M08222 | ICD-10-CM | Juvenile rheumatoid arthritis with systemic onset, left elbow |
| Rheum | JIA | M08229 | ICD-10-CM | Juvenile rheumatoid arthritis with systemic onset, unspecified elbow |
| Rheum | JIA | M08231 | ICD-10-CM | Juvenile rheumatoid arthritis with systemic onset, right wrist |
| Rheum | JIA | M08232 | ICD-10-CM | Juvenile rheumatoid arthritis with systemic onset, left wrist |
| Rheum | JIA | M08239 | ICD-10-CM | Juvenile rheumatoid arthritis with systemic onset, unspecified wrist |
| Rheum | JIA | M08241 | ICD-10-CM | Juvenile rheumatoid arthritis with systemic onset, right hand |
| Rheum | JIA | M08242 | ICD-10-CM | Juvenile rheumatoid arthritis with systemic onset, left hand |
| Rheum | JIA | M08249 | ICD-10-CM | Juvenile rheumatoid arthritis with systemic onset, unspecified hand |
| Rheum | JIA | M08251 | ICD-10-CM | Juvenile rheumatoid arthritis with systemic onset, right hip |
| Rheum | JIA | M08252 | ICD-10-CM | Juvenile rheumatoid arthritis with systemic onset, left hip |
| Rheum | JIA | M08259 | ICD-10-CM | Juvenile rheumatoid arthritis with systemic onset, unspecified hip |
| Rheum | JIA | M08261 | ICD-10-CM | Juvenile rheumatoid arthritis with systemic onset, right knee |
| Rheum | JIA | M08262 | ICD-10-CM | Juvenile rheumatoid arthritis with systemic onset, left knee |
| Rheum | JIA | M08269 | ICD-10-CM | Juvenile rheumatoid arthritis with systemic onset, unspecified knee |
| Rheum | JIA | M08271 | ICD-10-CM | Juvenile rheumatoid arthritis with systemic onset, right ankle and foot |
| Rheum | JIA | M08272 | ICD-10-CM | Juvenile rheumatoid arthritis with systemic onset, left ankle and foot |
| Rheum | JIA | M08279 | ICD-10-CM | Juvenile rheumatoid arthritis with systemic onset, unspecified ankle and foot |
| Rheum | JIA | M0828 | ICD-10-CM | Juvenile rheumatoid arthritis with systemic onset, vertebrae |
| Rheum | JIA | M0829 | ICD-10-CM | Juvenile rheumatoid arthritis with systemic onset, multiple sites |
| Rheum | JIA | M083 | ICD-10-CM | Juvenile rheumatoid polyarthritis (seronegative) |
| Rheum | JIA | M0840 | ICD-10-CM | Pauciarticular juvenile rheumatoid arthritis, unspecified site |
| Rheum | JIA | M08411 | ICD-10-CM | Pauciarticular juvenile rheumatoid arthritis, right shoulder |
| Rheum | JIA | M08412 | ICD-10-CM | Pauciarticular juvenile rheumatoid arthritis, left shoulder |
| Rheum | JIA | M08419 | ICD-10-CM | Pauciarticular juvenile rheumatoid arthritis, unspecified shoulder |
| Rheum | JIA | M08421 | ICD-10-CM | Pauciarticular juvenile rheumatoid arthritis, right elbow |
| Rheum | JIA | M08422 | ICD-10-CM | Pauciarticular juvenile rheumatoid arthritis, left elbow |
| Rheum | JIA | M08429 | ICD-10-CM | Pauciarticular juvenile rheumatoid arthritis, unspecified elbow |
| Rheum | JIA | M08431 | ICD-10-CM | Pauciarticular juvenile rheumatoid arthritis, right wrist |
| Rheum | JIA | M08432 | ICD-10-CM | Pauciarticular juvenile rheumatoid arthritis, left wrist |
| Rheum | JIA | M08439 | ICD-10-CM | Pauciarticular juvenile rheumatoid arthritis, unspecified wrist |
| Rheum | JIA | M08441 | ICD-10-CM | Pauciarticular juvenile rheumatoid arthritis, right hand |
| Rheum | JIA | M08442 | ICD-10-CM | Pauciarticular juvenile rheumatoid arthritis, left hand |
| Rheum | JIA | M08449 | ICD-10-CM | Pauciarticular juvenile rheumatoid arthritis, unspecified hand |
| Rheum | JIA | M08451 | ICD-10-CM | Pauciarticular juvenile rheumatoid arthritis, right hip |
| Rheum | JIA | M08452 | ICD-10-CM | Pauciarticular juvenile rheumatoid arthritis, left hip |
| Rheum | JIA | M08459 | ICD-10-CM | Pauciarticular juvenile rheumatoid arthritis, unspecified hip |
| Rheum | JIA | M08461 | ICD-10-CM | Pauciarticular juvenile rheumatoid arthritis, right knee |
| Rheum | JIA | M08462 | ICD-10-CM | Pauciarticular juvenile rheumatoid arthritis, left knee |
| Rheum | JIA | M08469 | ICD-10-CM | Pauciarticular juvenile rheumatoid arthritis, unspecified knee |
| Rheum | JIA | M08471 | ICD-10-CM | Pauciarticular juvenile rheumatoid arthritis, right ankle and foot |
| Rheum | JIA | M08472 | ICD-10-CM | Pauciarticular juvenile rheumatoid arthritis, left ankle and foot |
| Rheum | JIA | M08479 | ICD-10-CM | Pauciarticular juvenile rheumatoid arthritis, unspecified ankle and foot |
| Rheum | JIA | M0848 | ICD-10-CM | Pauciarticular juvenile rheumatoid arthritis, vertebrae |
| Rheum | JIA | M0880 | ICD-10-CM | Other juvenile arthritis, unspecified site |
| Rheum | JIA | M08811 | ICD-10-CM | Other juvenile arthritis, right shoulder |
| Rheum | JIA | M08812 | ICD-10-CM | Other juvenile arthritis, left shoulder |
| Rheum | JIA | M08819 | ICD-10-CM | Other juvenile arthritis, unspecified shoulder |
| Rheum | JIA | M08821 | ICD-10-CM | Other juvenile arthritis, right elbow |
| Rheum | JIA | M08822 | ICD-10-CM | Other juvenile arthritis, left elbow |
| Rheum | JIA | M08829 | ICD-10-CM | Other juvenile arthritis, unspecified elbow |
| Rheum | JIA | M08831 | ICD-10-CM | Other juvenile arthritis, right wrist |
| Rheum | JIA | M08832 | ICD-10-CM | Other juvenile arthritis, left wrist |
| Rheum | JIA | M08839 | ICD-10-CM | Other juvenile arthritis, unspecified wrist |
| Rheum | JIA | M08841 | ICD-10-CM | Other juvenile arthritis, right hand |
| Rheum | JIA | M08842 | ICD-10-CM | Other juvenile arthritis, left hand |
| Rheum | JIA | M08849 | ICD-10-CM | Other juvenile arthritis, unspecified hand |
| Rheum | JIA | M08851 | ICD-10-CM | Other juvenile arthritis, right hip |
| Rheum | JIA | M08852 | ICD-10-CM | Other juvenile arthritis, left hip |
| Rheum | JIA | M08859 | ICD-10-CM | Other juvenile arthritis, unspecified hip |
| Rheum | JIA | M08861 | ICD-10-CM | Other juvenile arthritis, right knee |
| Rheum | JIA | M08862 | ICD-10-CM | Other juvenile arthritis, left knee |
| Rheum | JIA | M08869 | ICD-10-CM | Other juvenile arthritis, unspecified knee |
| Rheum | JIA | M08871 | ICD-10-CM | Other juvenile arthritis, right ankle and foot |
| Rheum | JIA | M08872 | ICD-10-CM | Other juvenile arthritis, left ankle and foot |
| Rheum | JIA | M08879 | ICD-10-CM | Other juvenile arthritis, unspecified ankle and foot |
| Rheum | JIA | M0888 | ICD-10-CM | Other juvenile arthritis, vertebrae |
| Rheum | JIA | M0889 | ICD-10-CM | Other juvenile arthritis, multiple sites |
| Rheum | JIA | M0890 | ICD-10-CM | Juvenile arthritis, unspecified, unspecified site |
| Rheum | JIA | M08911 | ICD-10-CM | Juvenile arthritis, unspecified, right shoulder |
| Rheum | JIA | M08912 | ICD-10-CM | Juvenile arthritis, unspecified, left shoulder |
| Rheum | JIA | M08919 | ICD-10-CM | Juvenile arthritis, unspecified, unspecified shoulder |
| Rheum | JIA | M08921 | ICD-10-CM | Juvenile arthritis, unspecified, right elbow |
| Rheum | JIA | M08922 | ICD-10-CM | Juvenile arthritis, unspecified, left elbow |
| Rheum | JIA | M08929 | ICD-10-CM | Juvenile arthritis, unspecified, unspecified elbow |
| Rheum | JIA | M08931 | ICD-10-CM | Juvenile arthritis, unspecified, right wrist |
| Rheum | JIA | M08932 | ICD-10-CM | Juvenile arthritis, unspecified, left wrist |
| Rheum | JIA | M08939 | ICD-10-CM | Juvenile arthritis, unspecified, unspecified wrist |
| Rheum | JIA | M08941 | ICD-10-CM | Juvenile arthritis, unspecified, right hand |
| Rheum | JIA | M08942 | ICD-10-CM | Juvenile arthritis, unspecified, left hand |
| Rheum | JIA | M08949 | ICD-10-CM | Juvenile arthritis, unspecified, unspecified hand |
| Rheum | JIA | M08951 | ICD-10-CM | Juvenile arthritis, unspecified, right hip |
| Rheum | JIA | M08952 | ICD-10-CM | Juvenile arthritis, unspecified, left hip |
| Rheum | JIA | M08959 | ICD-10-CM | Juvenile arthritis, unspecified, unspecified hip |
| Rheum | JIA | M08961 | ICD-10-CM | Juvenile arthritis, unspecified, right knee |
| Rheum | JIA | M08962 | ICD-10-CM | Juvenile arthritis, unspecified, left knee |
| Rheum | JIA | M08969 | ICD-10-CM | Juvenile arthritis, unspecified, unspecified knee |
| Rheum | JIA | M08971 | ICD-10-CM | Juvenile arthritis, unspecified, right ankle and foot |
| Rheum | JIA | M08972 | ICD-10-CM | Juvenile arthritis, unspecified, left ankle and foot |
| Rheum | JIA | M08979 | ICD-10-CM | Juvenile arthritis, unspecified, unspecified ankle and foot |
| Rheum | JIA | M0898 | ICD-10-CM | Juvenile arthritis, unspecified, vertebrae |
| Rheum | JIA | M0899 | ICD-10-CM | Juvenile arthritis, unspecified, multiple sites |
| Rheum | RA | 7140 | ICD-9-CM | Rheumatoid arthritis |
| Rheum | RA | 7141 | ICD-9-CM | Felty's syndrome |
| Rheum | RA | 7142 | ICD-9-CM | Other rheumatoid arthritis with visceral or systemic involvement |
| Rheum | RA | 7144 | ICD-9-CM | Chronic postrheumatic arthropathy |
| Rheum | RA | M0500 | ICD-10-CM | Felty's syndrome, unspecified site |
| Rheum | RA | M05011 | ICD-10-CM | Felty's syndrome, right shoulder |
| Rheum | RA | M05012 | ICD-10-CM | Felty's syndrome, left shoulder |
| Rheum | RA | M05019 | ICD-10-CM | Felty's syndrome, unspecified shoulder |
| Rheum | RA | M05021 | ICD-10-CM | Felty's syndrome, right elbow |
| Rheum | RA | M05022 | ICD-10-CM | Felty's syndrome, left elbow |
| Rheum | RA | M05029 | ICD-10-CM | Felty's syndrome, unspecified elbow |
| Rheum | RA | M05031 | ICD-10-CM | Felty's syndrome, right wrist |
| Rheum | RA | M05032 | ICD-10-CM | Felty's syndrome, left wrist |
| Rheum | RA | M05039 | ICD-10-CM | Felty's syndrome, unspecified wrist |
| Rheum | RA | M05041 | ICD-10-CM | Felty's syndrome, right hand |
| Rheum | RA | M05042 | ICD-10-CM | Felty's syndrome, left hand |
| Rheum | RA | M05049 | ICD-10-CM | Felty's syndrome, unspecified hand |
| Rheum | RA | M05051 | ICD-10-CM | Felty's syndrome, right hip |
| Rheum | RA | M05052 | ICD-10-CM | Felty's syndrome, left hip |
| Rheum | RA | M05059 | ICD-10-CM | Felty's syndrome, unspecified hip |
| Rheum | RA | M05061 | ICD-10-CM | Felty's syndrome, right knee |
| Rheum | RA | M05062 | ICD-10-CM | Felty's syndrome, left knee |
| Rheum | RA | M05069 | ICD-10-CM | Felty's syndrome, unspecified knee |
| Rheum | RA | M05071 | ICD-10-CM | Felty's syndrome, right ankle and foot |
| Rheum | RA | M05072 | ICD-10-CM | Felty's syndrome, left ankle and foot |
| Rheum | RA | M05079 | ICD-10-CM | Felty's syndrome, unspecified ankle and foot |
| Rheum | RA | M0509 | ICD-10-CM | Felty's syndrome, multiple sites |
| Rheum | RA | M0510 | ICD-10-CM | Rheumatoid lung disease with rheumatoid arthritis of unspecified site |
| Rheum | RA | M05111 | ICD-10-CM | Rheumatoid lung disease with rheumatoid arthritis of right shoulder |
| Rheum | RA | M05112 | ICD-10-CM | Rheumatoid lung disease with rheumatoid arthritis of left shoulder |
| Rheum | RA | M05119 | ICD-10-CM | Rheumatoid lung disease with rheumatoid arthritis of unspecified shoulder |
| Rheum | RA | M05121 | ICD-10-CM | Rheumatoid lung disease with rheumatoid arthritis of right elbow |
| Rheum | RA | M05122 | ICD-10-CM | Rheumatoid lung disease with rheumatoid arthritis of left elbow |
| Rheum | RA | M05129 | ICD-10-CM | Rheumatoid lung disease with rheumatoid arthritis of unspecified elbow |
| Rheum | RA | M05131 | ICD-10-CM | Rheumatoid lung disease with rheumatoid arthritis of right wrist |
| Rheum | RA | M05132 | ICD-10-CM | Rheumatoid lung disease with rheumatoid arthritis of left wrist |
| Rheum | RA | M05139 | ICD-10-CM | Rheumatoid lung disease with rheumatoid arthritis of unspecified wrist |
| Rheum | RA | M05141 | ICD-10-CM | Rheumatoid lung disease with rheumatoid arthritis of right hand |
| Rheum | RA | M05142 | ICD-10-CM | Rheumatoid lung disease with rheumatoid arthritis of left hand |
| Rheum | RA | M05149 | ICD-10-CM | Rheumatoid lung disease with rheumatoid arthritis of unspecified hand |
| Rheum | RA | M05151 | ICD-10-CM | Rheumatoid lung disease with rheumatoid arthritis of right hip |
| Rheum | RA | M05152 | ICD-10-CM | Rheumatoid lung disease with rheumatoid arthritis of left hip |
| Rheum | RA | M05159 | ICD-10-CM | Rheumatoid lung disease with rheumatoid arthritis of unspecified hip |
| Rheum | RA | M05161 | ICD-10-CM | Rheumatoid lung disease with rheumatoid arthritis of right knee |
| Rheum | RA | M05162 | ICD-10-CM | Rheumatoid lung disease with rheumatoid arthritis of left knee |
| Rheum | RA | M05169 | ICD-10-CM | Rheumatoid lung disease with rheumatoid arthritis of unspecified knee |
| Rheum | RA | M05171 | ICD-10-CM | Rheumatoid lung disease with rheumatoid arthritis of right ankle and foot |
| Rheum | RA | M05172 | ICD-10-CM | Rheumatoid lung disease with rheumatoid arthritis of left ankle and foot |
| Rheum | RA | M05179 | ICD-10-CM | Rheumatoid lung disease with rheumatoid arthritis of unspecified ankle and foot |
| Rheum | RA | M0519 | ICD-10-CM | Rheumatoid lung disease with rheumatoid arthritis of multiple sites |
| Rheum | RA | M0520 | ICD-10-CM | Rheumatoid vasculitis with rheumatoid arthritis of unspecified site |
| Rheum | RA | M05211 | ICD-10-CM | Rheumatoid vasculitis with rheumatoid arthritis of right shoulder |
| Rheum | RA | M05212 | ICD-10-CM | Rheumatoid vasculitis with rheumatoid arthritis of left shoulder |
| Rheum | RA | M05219 | ICD-10-CM | Rheumatoid vasculitis with rheumatoid arthritis of unspecified shoulder |
| Rheum | RA | M05221 | ICD-10-CM | Rheumatoid vasculitis with rheumatoid arthritis of right elbow |
| Rheum | RA | M05222 | ICD-10-CM | Rheumatoid vasculitis with rheumatoid arthritis of left elbow |
| Rheum | RA | M05229 | ICD-10-CM | Rheumatoid vasculitis with rheumatoid arthritis of unspecified elbow |
| Rheum | RA | M05231 | ICD-10-CM | Rheumatoid vasculitis with rheumatoid arthritis of right wrist |
| Rheum | RA | M05232 | ICD-10-CM | Rheumatoid vasculitis with rheumatoid arthritis of left wrist |
| Rheum | RA | M05239 | ICD-10-CM | Rheumatoid vasculitis with rheumatoid arthritis of unspecified wrist |
| Rheum | RA | M05241 | ICD-10-CM | Rheumatoid vasculitis with rheumatoid arthritis of right hand |
| Rheum | RA | M05242 | ICD-10-CM | Rheumatoid vasculitis with rheumatoid arthritis of left hand |
| Rheum | RA | M05249 | ICD-10-CM | Rheumatoid vasculitis with rheumatoid arthritis of unspecified hand |
| Rheum | RA | M05251 | ICD-10-CM | Rheumatoid vasculitis with rheumatoid arthritis of right hip |
| Rheum | RA | M05252 | ICD-10-CM | Rheumatoid vasculitis with rheumatoid arthritis of left hip |
| Rheum | RA | M05259 | ICD-10-CM | Rheumatoid vasculitis with rheumatoid arthritis of unspecified hip |
| Rheum | RA | M05261 | ICD-10-CM | Rheumatoid vasculitis with rheumatoid arthritis of right knee |
| Rheum | RA | M05262 | ICD-10-CM | Rheumatoid vasculitis with rheumatoid arthritis of left knee |
| Rheum | RA | M05269 | ICD-10-CM | Rheumatoid vasculitis with rheumatoid arthritis of unspecified knee |
| Rheum | RA | M05271 | ICD-10-CM | Rheumatoid vasculitis with rheumatoid arthritis of right ankle and foot |
| Rheum | RA | M05272 | ICD-10-CM | Rheumatoid vasculitis with rheumatoid arthritis of left ankle and foot |
| Rheum | RA | M05279 | ICD-10-CM | Rheumatoid vasculitis with rheumatoid arthritis of unspecified ankle and foot |
| Rheum | RA | M0529 | ICD-10-CM | Rheumatoid vasculitis with rheumatoid arthritis of multiple sites |
| Rheum | RA | M0530 | ICD-10-CM | Rheumatoid heart disease with rheumatoid arthritis of unspecified site |
| Rheum | RA | M05311 | ICD-10-CM | Rheumatoid heart disease with rheumatoid arthritis of right shoulder |
| Rheum | RA | M05312 | ICD-10-CM | Rheumatoid heart disease with rheumatoid arthritis of left shoulder |
| Rheum | RA | M05319 | ICD-10-CM | Rheumatoid heart disease with rheumatoid arthritis of unspecified shoulder |
| Rheum | RA | M05321 | ICD-10-CM | Rheumatoid heart disease with rheumatoid arthritis of right elbow |
| Rheum | RA | M05322 | ICD-10-CM | Rheumatoid heart disease with rheumatoid arthritis of left elbow |
| Rheum | RA | M05329 | ICD-10-CM | Rheumatoid heart disease with rheumatoid arthritis of unspecified elbow |
| Rheum | RA | M05331 | ICD-10-CM | Rheumatoid heart disease with rheumatoid arthritis of right wrist |
| Rheum | RA | M05332 | ICD-10-CM | Rheumatoid heart disease with rheumatoid arthritis of left wrist |
| Rheum | RA | M05339 | ICD-10-CM | Rheumatoid heart disease with rheumatoid arthritis of unspecified wrist |
| Rheum | RA | M05341 | ICD-10-CM | Rheumatoid heart disease with rheumatoid arthritis of right hand |
| Rheum | RA | M05342 | ICD-10-CM | Rheumatoid heart disease with rheumatoid arthritis of left hand |
| Rheum | RA | M05349 | ICD-10-CM | Rheumatoid heart disease with rheumatoid arthritis of unspecified hand |
| Rheum | RA | M05351 | ICD-10-CM | Rheumatoid heart disease with rheumatoid arthritis of right hip |
| Rheum | RA | M05352 | ICD-10-CM | Rheumatoid heart disease with rheumatoid arthritis of left hip |
| Rheum | RA | M05359 | ICD-10-CM | Rheumatoid heart disease with rheumatoid arthritis of unspecified hip |
| Rheum | RA | M05361 | ICD-10-CM | Rheumatoid heart disease with rheumatoid arthritis of right knee |
| Rheum | RA | M05362 | ICD-10-CM | Rheumatoid heart disease with rheumatoid arthritis of left knee |
| Rheum | RA | M05369 | ICD-10-CM | Rheumatoid heart disease with rheumatoid arthritis of unspecified knee |
| Rheum | RA | M05371 | ICD-10-CM | Rheumatoid heart disease with rheumatoid arthritis of right ankle and foot |
| Rheum | RA | M05372 | ICD-10-CM | Rheumatoid heart disease with rheumatoid arthritis of left ankle and foot |
| Rheum | RA | M05379 | ICD-10-CM | Rheumatoid heart disease with rheumatoid arthritis of unspecified ankle and foot |
| Rheum | RA | M0539 | ICD-10-CM | Rheumatoid heart disease with rheumatoid arthritis of multiple sites |
| Rheum | RA | M0540 | ICD-10-CM | Rheumatoid myopathy with rheumatoid arthritis of unspecified site |
| Rheum | RA | M05411 | ICD-10-CM | Rheumatoid myopathy with rheumatoid arthritis of right shoulder |
| Rheum | RA | M05412 | ICD-10-CM | Rheumatoid myopathy with rheumatoid arthritis of left shoulder |
| Rheum | RA | M05419 | ICD-10-CM | Rheumatoid myopathy with rheumatoid arthritis of unspecified shoulder |
| Rheum | RA | M05421 | ICD-10-CM | Rheumatoid myopathy with rheumatoid arthritis of right elbow |
| Rheum | RA | M05422 | ICD-10-CM | Rheumatoid myopathy with rheumatoid arthritis of left elbow |
| Rheum | RA | M05429 | ICD-10-CM | Rheumatoid myopathy with rheumatoid arthritis of unspecified elbow |
| Rheum | RA | M05431 | ICD-10-CM | Rheumatoid myopathy with rheumatoid arthritis of right wrist |
| Rheum | RA | M05432 | ICD-10-CM | Rheumatoid myopathy with rheumatoid arthritis of left wrist |
| Rheum | RA | M05439 | ICD-10-CM | Rheumatoid myopathy with rheumatoid arthritis of unspecified wrist |
| Rheum | RA | M05441 | ICD-10-CM | Rheumatoid myopathy with rheumatoid arthritis of right hand |
| Rheum | RA | M05442 | ICD-10-CM | Rheumatoid myopathy with rheumatoid arthritis of left hand |
| Rheum | RA | M05449 | ICD-10-CM | Rheumatoid myopathy with rheumatoid arthritis of unspecified hand |
| Rheum | RA | M05451 | ICD-10-CM | Rheumatoid myopathy with rheumatoid arthritis of right hip |
| Rheum | RA | M05452 | ICD-10-CM | Rheumatoid myopathy with rheumatoid arthritis of left hip |
| Rheum | RA | M05459 | ICD-10-CM | Rheumatoid myopathy with rheumatoid arthritis of unspecified hip |
| Rheum | RA | M05461 | ICD-10-CM | Rheumatoid myopathy with rheumatoid arthritis of right knee |
| Rheum | RA | M05462 | ICD-10-CM | Rheumatoid myopathy with rheumatoid arthritis of left knee |
| Rheum | RA | M05469 | ICD-10-CM | Rheumatoid myopathy with rheumatoid arthritis of unspecified knee |
| Rheum | RA | M05471 | ICD-10-CM | Rheumatoid myopathy with rheumatoid arthritis of right ankle and foot |
| Rheum | RA | M05472 | ICD-10-CM | Rheumatoid myopathy with rheumatoid arthritis of left ankle and foot |
| Rheum | RA | M05479 | ICD-10-CM | Rheumatoid myopathy with rheumatoid arthritis of unspecified ankle and foot |
| Rheum | RA | M0549 | ICD-10-CM | Rheumatoid myopathy with rheumatoid arthritis of multiple sites |
| Rheum | RA | M0550 | ICD-10-CM | Rheumatoid polyneuropathy with rheumatoid arthritis of unspecified site |
| Rheum | RA | M05511 | ICD-10-CM | Rheumatoid polyneuropathy with rheumatoid arthritis of right shoulder |
| Rheum | RA | M05512 | ICD-10-CM | Rheumatoid polyneuropathy with rheumatoid arthritis of left shoulder |
| Rheum | RA | M05519 | ICD-10-CM | Rheumatoid polyneuropathy with rheumatoid arthritis of unspecified shoulder |
| Rheum | RA | M05521 | ICD-10-CM | Rheumatoid polyneuropathy with rheumatoid arthritis of right elbow |
| Rheum | RA | M05522 | ICD-10-CM | Rheumatoid polyneuropathy with rheumatoid arthritis of left elbow |
| Rheum | RA | M05529 | ICD-10-CM | Rheumatoid polyneuropathy with rheumatoid arthritis of unspecified elbow |
| Rheum | RA | M05531 | ICD-10-CM | Rheumatoid polyneuropathy with rheumatoid arthritis of right wrist |
| Rheum | RA | M05532 | ICD-10-CM | Rheumatoid polyneuropathy with rheumatoid arthritis of left wrist |
| Rheum | RA | M05539 | ICD-10-CM | Rheumatoid polyneuropathy with rheumatoid arthritis of unspecified wrist |
| Rheum | RA | M05541 | ICD-10-CM | Rheumatoid polyneuropathy with rheumatoid arthritis of right hand |
| Rheum | RA | M05542 | ICD-10-CM | Rheumatoid polyneuropathy with rheumatoid arthritis of left hand |
| Rheum | RA | M05549 | ICD-10-CM | Rheumatoid polyneuropathy with rheumatoid arthritis of unspecified hand |
| Rheum | RA | M05551 | ICD-10-CM | Rheumatoid polyneuropathy with rheumatoid arthritis of right hip |
| Rheum | RA | M05552 | ICD-10-CM | Rheumatoid polyneuropathy with rheumatoid arthritis of left hip |
| Rheum | RA | M05559 | ICD-10-CM | Rheumatoid polyneuropathy with rheumatoid arthritis of unspecified hip |
| Rheum | RA | M05561 | ICD-10-CM | Rheumatoid polyneuropathy with rheumatoid arthritis of right knee |
| Rheum | RA | M05562 | ICD-10-CM | Rheumatoid polyneuropathy with rheumatoid arthritis of left knee |
| Rheum | RA | M05569 | ICD-10-CM | Rheumatoid polyneuropathy with rheumatoid arthritis of unspecified knee |
| Rheum | RA | M05571 | ICD-10-CM | Rheumatoid polyneuropathy with rheumatoid arthritis of right ankle and foot |
| Rheum | RA | M05572 | ICD-10-CM | Rheumatoid polyneuropathy with rheumatoid arthritis of left ankle and foot |
| Rheum | RA | M05579 | ICD-10-CM | Rheumatoid polyneuropathy with rheumatoid arthritis of unspecified ankle and foot |
| Rheum | RA | M0559 | ICD-10-CM | Rheumatoid polyneuropathy with rheumatoid arthritis of multiple sites |
| Rheum | RA | M0560 | ICD-10-CM | Rheumatoid arthritis of unspecified site with involvement of other organs and systems |
| Rheum | RA | M05611 | ICD-10-CM | Rheumatoid arthritis of right shoulder with involvement of other organs and systems |
| Rheum | RA | M05612 | ICD-10-CM | Rheumatoid arthritis of left shoulder with involvement of other organs and systems |
| Rheum | RA | M05619 | ICD-10-CM | Rheumatoid arthritis of unspecified shoulder with involvement of other organs and systems |
| Rheum | RA | M05621 | ICD-10-CM | Rheumatoid arthritis of right elbow with involvement of other organs and systems |
| Rheum | RA | M05622 | ICD-10-CM | Rheumatoid arthritis of left elbow with involvement of other organs and systems |
| Rheum | RA | M05629 | ICD-10-CM | Rheumatoid arthritis of unspecified elbow with involvement of other organs and systems |
| Rheum | RA | M05631 | ICD-10-CM | Rheumatoid arthritis of right wrist with involvement of other organs and systems |
| Rheum | RA | M05632 | ICD-10-CM | Rheumatoid arthritis of left wrist with involvement of other organs and systems |
| Rheum | RA | M05639 | ICD-10-CM | Rheumatoid arthritis of unspecified wrist with involvement of other organs and systems |
| Rheum | RA | M05641 | ICD-10-CM | Rheumatoid arthritis of right hand with involvement of other organs and systems |
| Rheum | RA | M05642 | ICD-10-CM | Rheumatoid arthritis of left hand with involvement of other organs and systems |
| Rheum | RA | M05649 | ICD-10-CM | Rheumatoid arthritis of unspecified hand with involvement of other organs and systems |
| Rheum | RA | M05651 | ICD-10-CM | Rheumatoid arthritis of right hip with involvement of other organs and systems |
| Rheum | RA | M05652 | ICD-10-CM | Rheumatoid arthritis of left hip with involvement of other organs and systems |
| Rheum | RA | M05659 | ICD-10-CM | Rheumatoid arthritis of unspecified hip with involvement of other organs and systems |
| Rheum | RA | M05661 | ICD-10-CM | Rheumatoid arthritis of right knee with involvement of other organs and systems |
| Rheum | RA | M05662 | ICD-10-CM | Rheumatoid arthritis of left knee with involvement of other organs |
| Rheum | RA | M05669 | ICD-10-CM | Rheumatoid arthritis of unspecified knee with involvement of other organs |
| Rheum | RA | M05671 | ICD-10-CM | Rheumatoid arthritis of right ankle and foot with involvement of other organs and systems |
| Rheum | RA | M05672 | ICD-10-CM | Rheumatoid arthritis of left ankle and foot with involvement of other organs and systems |
| Rheum | RA | M05679 | ICD-10-CM | Rheumatoid arthritis of unspecified ankle and foot with involvement of other organs and systems |
| Rheum | RA | M0569 | ICD-10-CM | Rheumatoid arthritis of multiple sites with involvement of other organ |
| Rheum | RA | M0570 | ICD-10-CM | Rheumatoid arthritis with rheumatoid factor of unspecified site without organ or systems involvement |
| Rheum | RA | M05711 | ICD-10-CM | Rheumatoid arthritis with rheumatoid factor of right shoulder without organ or systems involvement |
| Rheum | RA | M05712 | ICD-10-CM | Rheumatoid arthritis with rheumatoid factor of left shoulder without organ or systems involvement |
| Rheum | RA | M05719 | ICD-10-CM | Rheumatoid arthritis with rheumatoid factor of unspecified shoulder without organ or systems involvement |
| Rheum | RA | M05721 | ICD-10-CM | Rheumatoid arthritis with rheumatoid factor of right elbow without organ or systems involvement |
| Rheum | RA | M05722 | ICD-10-CM | Rheumatoid arthritis with rheumatoid factor of left elbow without organ or systems involvement |
| Rheum | RA | M05729 | ICD-10-CM | Rheumatoid arthritis with rheumatoid factor of unspecified elbow without organ or systems involvement |
| Rheum | RA | M05731 | ICD-10-CM | Rheumatoid arthritis with rheumatoid factor of right wrist without organ or systems involvement |
| Rheum | RA | M05732 | ICD-10-CM | Rheumatoid arthritis with rheumatoid factor of left wrist without organ or systems involvement |
| Rheum | RA | M05739 | ICD-10-CM | Rheumatoid arthritis with rheumatoid factor of unspecified wrist without organ or systems involvement |
| Rheum | RA | M05741 | ICD-10-CM | Rheumatoid arthritis with rheumatoid factor of right hand without organ or systems involvement |
| Rheum | RA | M05742 | ICD-10-CM | Rheumatoid arthritis with rheumatoid factor of left hand without organ or systems involvement |
| Rheum | RA | M05749 | ICD-10-CM | Rheumatoid arthritis with rheumatoid factor of unspecified hand without organ or systems involvement |
| Rheum | RA | M05751 | ICD-10-CM | Rheumatoid arthritis with rheumatoid factor of right hip without organ or systems involvement |
| Rheum | RA | M05752 | ICD-10-CM | Rheumatoid arthritis with rheumatoid factor of left hip without organ or systems involvement |
| Rheum | RA | M05759 | ICD-10-CM | Rheumatoid arthritis with rheumatoid factor of unspecified hip without organ or systems involvement |
| Rheum | RA | M05761 | ICD-10-CM | Rheumatoid arthritis with rheumatoid factor of right knee without organ or systems involvement |
| Rheum | RA | M05762 | ICD-10-CM | Rheumatoid arthritis with rheumatoid factor of left knee without organ or systems involvement |
| Rheum | RA | M05769 | ICD-10-CM | Rheumatoid arthritis with rheumatoid factor of unspecified knee without organ or systems involvement |
| Rheum | RA | M05771 | ICD-10-CM | Rheumatoid arthritis with rheumatoid factor of right ankle and foot without organ or systems involvement |
| Rheum | RA | M05772 | ICD-10-CM | Rheumatoid arthritis with rheumatoid factor of left ankle and foot without organ or systems involvement |
| Rheum | RA | M05779 | ICD-10-CM | Rheumatoid arthritis with rheumatoid factor of unspecified ankle and foot without organ or systems involvement |
| Rheum | RA | M0579 | ICD-10-CM | Rheumatoid arthritis with rheumatoid factor of multiple sites without organ or systems involvement |
| Rheum | RA | M0580 | ICD-10-CM | Other rheumatoid arthritis with rheumatoid factor of unspecified site |
| Rheum | RA | M05811 | ICD-10-CM | Other rheumatoid arthritis with rheumatoid factor of right shoulder |
| Rheum | RA | M05812 | ICD-10-CM | Other rheumatoid arthritis with rheumatoid factor of left shoulder |
| Rheum | RA | M05819 | ICD-10-CM | Other rheumatoid arthritis with rheumatoid factor of unspecified shoulder |
| Rheum | RA | M05821 | ICD-10-CM | Other rheumatoid arthritis with rheumatoid factor of right elbow |
| Rheum | RA | M05822 | ICD-10-CM | Other rheumatoid arthritis with rheumatoid factor of left elbow |
| Rheum | RA | M05829 | ICD-10-CM | Other rheumatoid arthritis with rheumatoid factor of unspecified elbow |
| Rheum | RA | M05831 | ICD-10-CM | Other rheumatoid arthritis with rheumatoid factor of right wrist |
| Rheum | RA | M05832 | ICD-10-CM | Other rheumatoid arthritis with rheumatoid factor of left wrist |
| Rheum | RA | M05839 | ICD-10-CM | Other rheumatoid arthritis with rheumatoid factor of unspecified wrist |
| Rheum | RA | M05841 | ICD-10-CM | Other rheumatoid arthritis with rheumatoid factor of right hand |
| Rheum | RA | M05842 | ICD-10-CM | Other rheumatoid arthritis with rheumatoid factor of left hand |
| Rheum | RA | M05849 | ICD-10-CM | Other rheumatoid arthritis with rheumatoid factor of unspecified hand |
| Rheum | RA | M05851 | ICD-10-CM | Other rheumatoid arthritis with rheumatoid factor of right hip |
| Rheum | RA | M05852 | ICD-10-CM | Other rheumatoid arthritis with rheumatoid factor of left hip |
| Rheum | RA | M05859 | ICD-10-CM | Other rheumatoid arthritis with rheumatoid factor of unspecified hip |
| Rheum | RA | M05861 | ICD-10-CM | Other rheumatoid arthritis with rheumatoid factor of right knee |
| Rheum | RA | M05862 | ICD-10-CM | Other rheumatoid arthritis with rheumatoid factor of left knee |
| Rheum | RA | M05869 | ICD-10-CM | Other rheumatoid arthritis with rheumatoid factor of unspecified knee |
| Rheum | RA | M05871 | ICD-10-CM | Other rheumatoid arthritis with rheumatoid factor of right ankle and foot |
| Rheum | RA | M05872 | ICD-10-CM | Other rheumatoid arthritis with rheumatoid factor of left ankle and foot |
| Rheum | RA | M05879 | ICD-10-CM | Other rheumatoid arthritis with rheumatoid factor of unspecified ankle and |
| Rheum | RA | M0589 | ICD-10-CM | Other rheumatoid arthritis with rheumatoid factor of multiple sites |
| Rheum | RA | M059 | ICD-10-CM | Rheumatoid arthritis with rheumatoid factor, unspecified |
| Rheum | RA | M0600 | ICD-10-CM | Rheumatoid arthritis without rheumatoid factor, unspecified site |
| Rheum | RA | M06011 | ICD-10-CM | Rheumatoid arthritis without rheumatoid factor, right shoulder |
| Rheum | RA | M06012 | ICD-10-CM | Rheumatoid arthritis without rheumatoid factor, left shoulder |
| Rheum | RA | M06019 | ICD-10-CM | Rheumatoid arthritis without rheumatoid factor, unspecified shoulder |
| Rheum | RA | M06021 | ICD-10-CM | Rheumatoid arthritis without rheumatoid factor, right elbow |
| Rheum | RA | M06022 | ICD-10-CM | Rheumatoid arthritis without rheumatoid factor, left elbow |
| Rheum | RA | M06029 | ICD-10-CM | Rheumatoid arthritis without rheumatoid factor, unspecified elbow |
| Rheum | RA | M06031 | ICD-10-CM | Rheumatoid arthritis without rheumatoid factor, right wrist |
| Rheum | RA | M06032 | ICD-10-CM | Rheumatoid arthritis without rheumatoid factor, left wrist |
| Rheum | RA | M06039 | ICD-10-CM | Rheumatoid arthritis without rheumatoid factor, unspecified wrist |
| Rheum | RA | M06041 | ICD-10-CM | Rheumatoid arthritis without rheumatoid factor, right hand |
| Rheum | RA | M06042 | ICD-10-CM | Rheumatoid arthritis without rheumatoid factor, left hand |
| Rheum | RA | M06049 | ICD-10-CM | Rheumatoid arthritis without rheumatoid factor, unspecified hand |
| Rheum | RA | M06051 | ICD-10-CM | Rheumatoid arthritis without rheumatoid factor, right hip |
| Rheum | RA | M06052 | ICD-10-CM | Rheumatoid arthritis without rheumatoid factor, left hip |
| Rheum | RA | M06059 | ICD-10-CM | Rheumatoid arthritis without rheumatoid factor, unspecified hip |
| Rheum | RA | M06061 | ICD-10-CM | Rheumatoid arthritis without rheumatoid factor, right knee |
| Rheum | RA | M06062 | ICD-10-CM | Rheumatoid arthritis without rheumatoid factor, left knee |
| Rheum | RA | M06069 | ICD-10-CM | Rheumatoid arthritis without rheumatoid factor, unspecified knee |
| Rheum | RA | M06071 | ICD-10-CM | Rheumatoid arthritis without rheumatoid factor, right ankle and foot |
| Rheum | RA | M06072 | ICD-10-CM | Rheumatoid arthritis without rheumatoid factor, left ankle and foot |
| Rheum | RA | M06079 | ICD-10-CM | Rheumatoid arthritis without rheumatoid factor, unspecified ankle and foot |
| Rheum | RA | M0608 | ICD-10-CM | Rheumatoid arthritis without rheumatoid factor, vertebrae |
| Rheum | RA | M0609 | ICD-10-CM | Rheumatoid arthritis without rheumatoid factor, multiple sites |
| Rheum | RA | M0680 | ICD-10-CM | Other specified rheumatoid arthritis, unspecified site |
| Rheum | RA | M06811 | ICD-10-CM | Other specified rheumatoid arthritis, right shoulder |
| Rheum | RA | M06812 | ICD-10-CM | Other specified rheumatoid arthritis, left shoulder |
| Rheum | RA | M06819 | ICD-10-CM | Other specified rheumatoid arthritis, unspecified shoulder |
| Rheum | RA | M06821 | ICD-10-CM | Other specified rheumatoid arthritis, right elbow |
| Rheum | RA | M06822 | ICD-10-CM | Other specified rheumatoid arthritis, left elbow |
| Rheum | RA | M06829 | ICD-10-CM | Other specified rheumatoid arthritis, unspecified elbow |
| Rheum | RA | M06831 | ICD-10-CM | Other specified rheumatoid arthritis, right wrist |
| Rheum | RA | M06832 | ICD-10-CM | Other specified rheumatoid arthritis, left wrist |
| Rheum | RA | M06839 | ICD-10-CM | Other specified rheumatoid arthritis, unspecified wrist |
| Rheum | RA | M06841 | ICD-10-CM | Other specified rheumatoid arthritis, right hand |
| Rheum | RA | M06842 | ICD-10-CM | Other specified rheumatoid arthritis, left hand |
| Rheum | RA | M06849 | ICD-10-CM | Other specified rheumatoid arthritis, unspecified hand |
| Rheum | RA | M06851 | ICD-10-CM | Other specified rheumatoid arthritis, right hip |
| Rheum | RA | M06852 | ICD-10-CM | Other specified rheumatoid arthritis, left hip |
| Rheum | RA | M06859 | ICD-10-CM | Other specified rheumatoid arthritis, unspecified hip |
| Rheum | RA | M06861 | ICD-10-CM | Other specified rheumatoid arthritis, right knee |
| Rheum | RA | M06862 | ICD-10-CM | Other specified rheumatoid arthritis, left knee |
| Rheum | RA | M06869 | ICD-10-CM | Other specified rheumatoid arthritis, unspecified knee |
| Rheum | RA | M06871 | ICD-10-CM | Other specified rheumatoid arthritis, right ankle and foot |
| Rheum | RA | M06872 | ICD-10-CM | Other specified rheumatoid arthritis, left ankle and foot |
| Rheum | RA | M06879 | ICD-10-CM | Other specified rheumatoid arthritis, unspecified ankle and foot |
| Rheum | RA | M0688 | ICD-10-CM | Other specified rheumatoid arthritis, vertebrae |
| Rheum | RA | M0689 | ICD-10-CM | Other specified rheumatoid arthritis, multiple sites |
| Rheum | RA | M069 | ICD-10-CM | Rheumatoid arthritis, unspecified |
| Rheum | SLE | 7100 | ICD-9-CM | Systemic lupus erythematosus |
| Rheum | SLE | M3210 | ICD-10-CM | Systemic lupus erythematosus, organ or system involvement unspecified |
| Rheum | SLE | M3211 | ICD-10-CM | Endocarditis in systemic lupus erythematosus |
| Rheum | SLE | M3212 | ICD-10-CM | Pericarditis in systemic lupus erythematosus |
| Rheum | SLE | M3213 | ICD-10-CM | Lung involvement in systemic lupus erythematosus |
| Rheum | SLE | M3214 | ICD-10-CM | Glomerular disease in systemic lupus erythematosus |
| Rheum | SLE | M3215 | ICD-10-CM | Tubulo-interstitial nephropathy in systemic lupus erythematosus |
| Rheum | SLE | M3219 | ICD-10-CM | Other organ or system involvement in systemic lupus erythematosus |
| Rheum | SLE | M328 | ICD-10-CM | Other forms of systemic lupus erythematosus |
| Rheum | SLE | M329 | ICD-10-CM | Systemic lupus erythematosus, unspecified |

1. Lo Re, et. al Validity of ICD-10-CM Diagnoses to Identify Hospitalizations for Serious Infections Among Patients Treated With Biologic Therapies (2020)

**Table 3: Presenting Symptoms/Status at time of SAMPLE COLLECTION**

Unless otherwise noted, variables in this table are assessed +/- 14 days from the index date; or +/- 14 days from the serology date, unless otherwise specified.

| **Variable** | **Algorithm / Definitions** | **Setting^1^** | **Source** | **Reference(s)** |
| --- | --- | --- | --- | --- |
| Pregnant (assess up to and including 40 weeks prior to index date) | ICD-10-CM: O00*-O92*, O94*-O9A  CPT/HCPCS codes: 59426, 59620, 59866, 59897, 76801, 76814, 76815, 76828, 80055, 85461, 99500, 59015, 59325, 59425, 59515, 76821, 76825, 76826, 76941, 76945, 76946, 85460, 88235, 99478, 99479, 59020, 59025, 59030, 59400, 59409, 59510, 59514, 59610, 59614, 59618, 59622, 76802, 76812, 76818, 76819, 83661, 99300, 59070, 59320, 76805, 76810, 76816, 76820, 76827, 82143, 83033, 0502F, 59000, 59001, 59410, 59612, 76811, 76813, 76817, 82731, 83663, 83664, 84163, 88267, 99480, 01958, 01960, 01967, 01961, 01962, 01968, 01969 | IP, OP, ED, or GL | Claims or EHR |  |
| Fever | ICD-10-CM admitting diagnosis: R50.9 | IP, OP, ED, or GL | Claims or EHR |  |
| Headache | ICD-10-CM admitting diagnosis: R51 | IP, OP, ED, or GL | Claims or EHR |  |
| Diarrhea | ICD-10-CM admitting diagnosis: K59.1, R19.7 | IP, OP, ED, or GL | Claims or EHR |  |
| Cough | ICD-10-CM admitting diagnosis: R05 | IP, OP, ED, or GL | Claims or EHR |  |
| Delirium/encephalopathy | ICD-10-CM admitting diagnosis: F05*, A81.2*, E51.2*, G04.30*, G04.31*, G04.32*, G04.39*, G92**, G93.40*, G93.41*, G93.49*, I67.3*, I67.4*, I67.83*, J10.81*, J11.81*, P91.60*, P91.61*, P91.62*, P91.63* | IP, OP, ED, or GL | Claims or EHR | [Kim et al, 2017](https://onlinelibrary.wiley.com/doi/abs/10.1002/pds.4226) |
| Chest pain | ICD-10-CM admitting diagnosis: R07.1, R07.8, R07.89, R07.9 | IP, OP, ED, or GL | Claims or EHR |  |
| Sore throat | ICD-10-CM admitting diagnosis: J02.9 | IP, OP, ED, or GL | Claims or EHR |  |
| Pneumonia | ICD-10-CM admitting diagnosis: J18.1, J18.9, J18.8, J18.0, J85.1, J12.89, J12.9, J12.81 | IP, OP, ED, or GL | Claims or EHR |  |
| Shortness of breath and related | ICD-10-CM admitting diagnosis: R09.02, R06.0*, R06.02, R06.2 | IP, OP, ED, or GL | Claims or EHR |  |
| Acute bronchitis | ICD-10-CM admitting diagnosis: J20.8 | IP, OP, ED, or GL | Claims or EHR |  |
| Other acute respiratory infection | ICD-10-CM admitting diagnosis: J06.9, J22 | IP, OP, ED, or GL | Claims or EHR |  |
| Acute respiratory distress, arrest, or failure | ICD-10-CM admitting diagnosis: J80, J96.0, J96.00, J96.01, J96.02, J96.2, J96.20, J96.21, J96.22, R09.2 | IP, OP, ED, or GL | Claims or EHR |  |
| Any cardiovascular condition | ICD-10-CM admitting diagnosis: I00*-I99* | IP, OP, ED, or GL | Claims or EHR | [Nyström et al, 2017](https://www.sciencedirect.com/science/article/pii/S0168822716304429#s0095) |
| Any renal condition | ICD-10-CM admitting diagnosis: N00*-N27*, N99.0, Z49.0*– Z49.2*, Z94.0*, Z99.2 | IP, OP, ED, or GL | Claims or EHR |  |
| …Influenza (lab confirmed) | Lab confirmed influenza A, B | IP, OP, ED, or GL | Claims or EHR |  |
| …RSV (lab confirmed) | Lab confirmed RSV | IP, OP, ED, or GL | Claims or EHR |  |
| Other antivirals (not HCQ, remdesivir) | NDC Generic Name: *abacavir*, *acyclovir *, *aciclovir*, *adefovir*, *amantadine*, *amprenavir*, *atazanavir*, *baloxavir marboxil *, *bictegravir*, *boceprevir*, *cidofovir*, *cobicistat *, *daclatasvir*, *darunavir*, *delavirdine*, *danoprevir*, *didanosine*, *docosanol*, *dolutegravir*, *doravirine*, *edoxudine*, *efavirenz*, *elvitegravir*, *emtricitabine*, *emtricitabine/tenofovir*, *enfuvirtide*, *entecavir*, *etravirine*, *famciclovir*, *favipiravir*, *fomivirsen*, *fosamprenavir*, *foscarnet*, *fosfonat*, *ganciclovir *, *galidesivir*, *ibalizumab *, *idoxuridine*, *imiquimod*, *inosine pranobex*, *indinavir*, *interferon alfa*, *interferon beta*, *interferon*, *lamivudine*, *letermovir*, *lopinavir*, *maraviroc*, *methisazone*, *moroxydine*, *nelfinavir*, *nevirapine*, *nexavir*, *nitazoxanide*, *oseltamivir*, *peginterferon alfa-2a*, *peginterferon alfa-2b*, *penciclovir*, *peramivir *, *pleconaril*, *podophyllotoxin*, *raltegravir*, *rilpivirine *, *rimantadine*, *ritonavir*, *rintatolimod*, *saquinavir*, *simeprevir*, *sofosbuvir*, *stavudine*, *taribavirin*, *telaprevir*, *telbivudine*, *tenofovir alafenamide*, *tenofovir disoproxil*, *tenofovir*, *tipranavir*, *trifluridine*, *tromantadine*, *umifenovir *, *valaciclovir *, *valganciclovir*, *vicriviroc*, *vidarabine*, *viramidine*, *zalcitabine*, *zanamivir*, *zidovudine*  Chargemaster Vendor Charge Code: *abacavir*, *acyclovir *, *aciclovir*, *adefovir*, *amantadine*, *amprenavir*, *atazanavir*, *baloxavir marboxil *, *bictegravir*, *boceprevir*, *cidofovir*, *cobicistat *, *daclatasvir*, *darunavir*, *delavirdine*, *danoprevir*, *didanosine*, *docosanol*, *dolutegravir*, *doravirine*, *edoxudine*, *efavirenz*, *elvitegravir*, *emtricitabine*, *emtricitabine/tenofovir*, *enfuvirtide*, *entecavir*, *etravirine*, *famciclovir*, *favipiravir*, *fomivirsen*, *fosamprenavir*, *foscarnet*, *fosfonat*, *ganciclovir *, *galidesivir*, *ibalizumab *, *idoxuridine*, *imiquimod*, *inosine pranobex*, *indinavir*, *interferon alfa*, *interferon beta*, *interferon*, *lamivudine*, *letermovir*, *lopinavir*, *maraviroc*, *methisazone*, *moroxydine*, *nelfinavir*, *nevirapine*, *nexavir*, *nitazoxanide*, *oseltamivir*, *peginterferon alfa-2a*, *peginterferon alfa-2b*, *penciclovir*, *peramivir *, *pleconaril*, *podophyllotoxin*, *raltegravir*, *rilpivirine *, *rimantadine*, *ritonavir*, *rintatolimod*, *saquinavir*, *simeprevir*, *sofosbuvir*, *stavudine*, *taribavirin*, *telaprevir*, *telbivudine*, *tenofovir alafenamide*, *tenofovir disoproxil*, *tenofovir*, *tipranavir*, *trifluridine*, *tromantadine*, *umifenovir *, *valaciclovir *, *valganciclovir*, *vicriviroc*, *vidarabine*, *viramidine*, *zalcitabine*, *zanamivir*, *zidovudine*, *Ziagen*, *Sitavig*, *Zovirax*, *Preveon*, *Hepsera*, *Gocovri*, *Agenerase*, *Reyataz*, *Xofluza*, *Biktarvy*, *Victrelis*, *Visitide*, *Tybost*, *Daklinza*, *Prezista*, *Rescriptor*, *Ganovo*, *Videx*, *Abreva*, *Dovato*, *Tivicay*, *Pifeltro*, *Sustiva*, *Symfi*, *Vitekta*, *Descovy*, *Truvada*, *Atripla*, *Biktarvy*, *Fuzeon*, *Baraclude*, *Intelence*, *Famvir*, *Avigan*, *Vitravene*, *Lexiva*, *Foscavir*, *Cytovene*, *Trogarzo*, *Dendrid*, *Herplex*, *Zyclara*, *Aldara*, *Imunovir*, *Crixivan*, *Intron A*, *Rebif*, *Roferon-A*, *Betaseron*, *Extavia*, *Avonex*, *Interfergen*, *Plegridy*, *Actimmune*, *Alferon*, *Combivir*, *Prevymis*, *Kaletra*, *Selzentry*, *Celsentri*, *Tapazole*, *Viracept*, *Viramune*, *Kutapressin*, *Alinia*, *Tamiflu*, *Pegasys*, *PegIntron*, *Pegridy*, *Denavir*, *Rapivab*, *Picovir*, *Condylox*, *Isentress*, *Edurant*, *Rebetol*, *Flumadine*, *Novir*, *Ampligen*, *Invirase*, *Fortovase*, *Olysio*, *Sovaldi*, *Zerit*, *Incivek*, *Incivo*, *Tyzeka*, *Odefsey*, *Complera*, *Vemlidy*, *Viread*, *Aptivus*, *Viroptic*, *Viru-Merz*, *Arbidol*, *Valtrex*, *Valcyte*, *Vira-A*, *Hivid*, *Relenza*, *Retrovir*, *Epzicom*, *Stribild*, *Zepatier*, *Dovato*, *Trizivir*, *Mavyret*, *Harvoni*, *Triumeq*, *Epclusa*, *Symtuza*, *Genvoya*, *Viekira*, *Juluca*, *Combivir*, *Prezcobix*, *AccessPak*, *Cimduo*, *Delstrigo*, *Evotaz*, *Technivie*, *Temixys*, *Vosevi* | IP, OP, ED, or GL | Claims or EHR |  |
| Known exposure to COVID-19 | ICD-10-CM Z20.828 (Contact with and (suspected) exposure to other viral communicable diseases) is used as a proxy for known exposure to COVID-19 in the baseline period. | IP, OP, ED, or GL | Claims or EHR |  |

1. IP=Inpatient; OP=Outpatient; ED=Emergency Department; GL=Group Living Facility
